# Recent Advances in Metasurfaces: From THz Biosensing to Microwave Wireless Communications

**DOI:** 10.34133/research.0820

**Published:** 2025-08-29

**Authors:** Yue Wang, Xiang Zhang, Yuxiang Wang, Yunfei Liu, Jiaxue Li, Xiangdong Chen, Zijian Cui, Shah Nawaz Burokur, Jingdi Zhang, Xiaoguang Zhao, Kuang Zhang, Zheng You

**Affiliations:** ^1^ Key Laboratory of Ultrafast Photoelectric and Terahertz Science in Shaanxi, Xi’an University of Technology, Xi’an, China.; ^2^ Department of Microwave Engineering, Harbin Institute of Technology, Harbin, China.; ^3^ State Key Laboratory of Precision Measurement Technology and Instruments, Department of Precision Instruments, Tsinghua University, Beijing, China.; ^4^ LEME, Univ Paris Nanterre, 92410 Ville d’Avray, France.; ^5^ Hong Kong University of Science and Technology, Hong Kong, China.

## Abstract

In recent years, important progress has been made in the field of biosensing and wireless communications by using metamaterials and metasurfaces. These technologies enable efficient manipulation of electromagnetic waves through judiciously designed subwavelength structural units. This review begins by focusing on the design and optimization of terahertz metasurface sensors, emphasizing their unique advantages in biomedical diagnostics. It explores key technical challenges, such as material selection, device integration, and development of robust sensor for surface-specific modifications. Furthermore, the review discusses how metasurfaces, particularly as reconfigurable intelligent surfaces, dynamically modulate electromagnetic wave propagation in the microwave communications domain to enhance signal quality, improve communication efficiency, and showcase their potential in 5G and future 6G technologies. Finally, a comprehensive overview is provided regarding the challenges and future research directions for metamaterial and metasurface technologies in both biosensing and wireless communications, with the ultimate goal of promoting their applications in point-of-care devices and efficient communication systems.

## Introduction

Metamaterials and metasurfaces have revolutionized the way we control electromagnetic waves. Engineered at subwavelength scales, these artificial electromagnetic materials enable electromagnetic functionalities far beyond those offered by natural materials [1–9]. Metasurfaces—2-dimensional (2D) analogs of metamaterials—combine compactness, low-loss operation, and exceptional design freedom, making them an indispensable platform across disciplines [10–16]. From molecular-scale terahertz (THz) biosensing to dynamic microwave communications, metasurfaces are bridging the gap between fundamental photonics and real-world technologies, offering transformative potential in both science and industry [17].

**Fig. 1.**
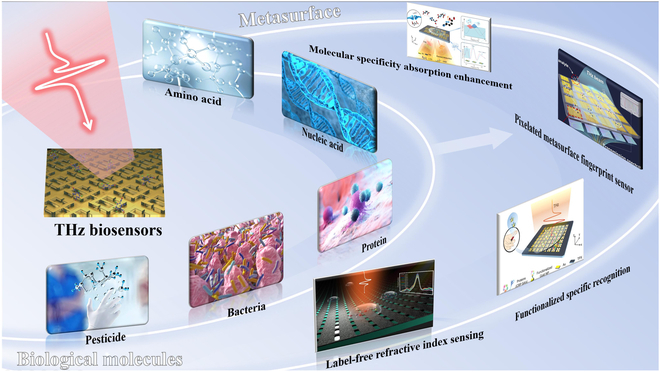
Different kinds of THz biosensors with different materials and structures.

THz waves refer to electromagnetic waves that lie between microwaves and infrared waves, within the frequency range of 0.1 to 10 THz. Unlike electromagnetic waves in other bands (such as x-rays and ultraviolet rays), THz waves are non-ionizing, with photon energy at the meV level, millions of times lower than x-rays, which do not damage the molecular structure of biological samples [18,19]. With the advances in THz wave technology, trace biochemistry substance detection based on THz biological effects has emerged as a promising research direction [20–22]. For example, trace biological sample detection based on THz bioeffects can effectively reveal the health status and pathological information of molecules, cells, or tissues within living organisms [23,24]. This method also ensures good biological safety. In particular, with the development of THz metasurface technology, it not only overcomes the mismatch between trace biological molecules and THz wavelengths but also enables precise control over electromagnetic wave propagation [25–29]. The optically controlled THz metasurface was initiated to achieve ultrafast switchable sensing functionalities [30]. This innovative study furnishes a promising new paradigm of constructing reliable and flexible THz metasurfaces with fast switchable multi-functions. It enhances light–matter interactions, achieving highly sensitive detection of trace biological samples, demonstrating significant potential, especially in molecular research and disease diagnosis, as shown in Fig. 1.

Many studies have demonstrated that water exhibits an exceptionally high absorption coefficient for THz waves [31–34]. For example, at approximately 1 THz, the absorption coefficient of liquid water can reach several hundred cm^−1^. This results in pronounced absorption features in water-contained samples within the THz range, enabling the analysis of moisture content and dynamic properties in biological samples using THz absorption spectroscopy. Since tumor cells and normal cells differ significantly in water content and tissue density, hydration status can substantially influence the propagation characteristics of THz waves, making it a valuable parameter for early cancer screening [35]. The periodic structural cells of THz metasurfaces, such as metallic ring resonators and slit structures, can induce phenomena like surface plasmon resonance (SPR) or bound states in the continuum (BIC). These phenomena generate strong localized electric field enhancements, enabling THz metasurface sensors to efficiently detect and differentiate between normal and cancerous cells [36]. Such capabilities are critical for early cancer diagnosis and tumor identification. Additionally, biological molecules, such as proteins, DNA, and lipids, exhibit characteristic absorption peaks in the THz range, corresponding to their vibrational and rotational modes. When THz waves interact with biological samples, specific frequencies are absorbed by the molecules, altering the transmission or reflection properties of the waves [37]. By analyzing these changes, valuable information about the chemical composition and structure of biological molecules can be obtained. Through the design of THz metasurfaces to amplify light–matter interactions, significant signal changes can be achieved, enabling the high-sensitivity detection of trace biological samples. For instance, in inflammation and infection detection, THz metasurfaces can monitor changes in cell morphology or variations in the concentration of proteins, nucleic acids, and other biomolecules, facilitating the identification of disease biomarkers for early diagnosis. The ability of THz metasurfaces to control electromagnetic waves and enhance localized electric fields, as exemplified by THz metasensors, is a key factor in achieving high-sensitivity biosensing [38].

Driven by the increasing demands of biosensing, THz metasurfaces have advanced to achieve key functions including label-free refractive index sensing, functionalized specific recognition, and pixelated metasurface fingerprint reconstruction [39–42]. Correspondingly, several typical material platforms have been developed, including metal-based metasurface sensors, carbon-based metasurface sensors, and metasurface sensors based on phase change functional materials such as liquid crystals and vanadium oxides [43–46]. Each represents a different material design and performance optimization strategy, with unique advantages in controlling THz waves: For instance, metal-based metasurfaces support SPR, dielectric materials provide low loss and high-quality factors, while graphene and carbon nanotubes (CNTs) offer dynamic tunability and optical anisotropy. By leveraging these material-specific properties, THz metasurfaces enable high-sensitivity detection and multifunctional capabilities for analyzing biological samples, showing significant promise for biomedical detection, disease diagnosis, and life science research [47–51].

**Fig. 2.**
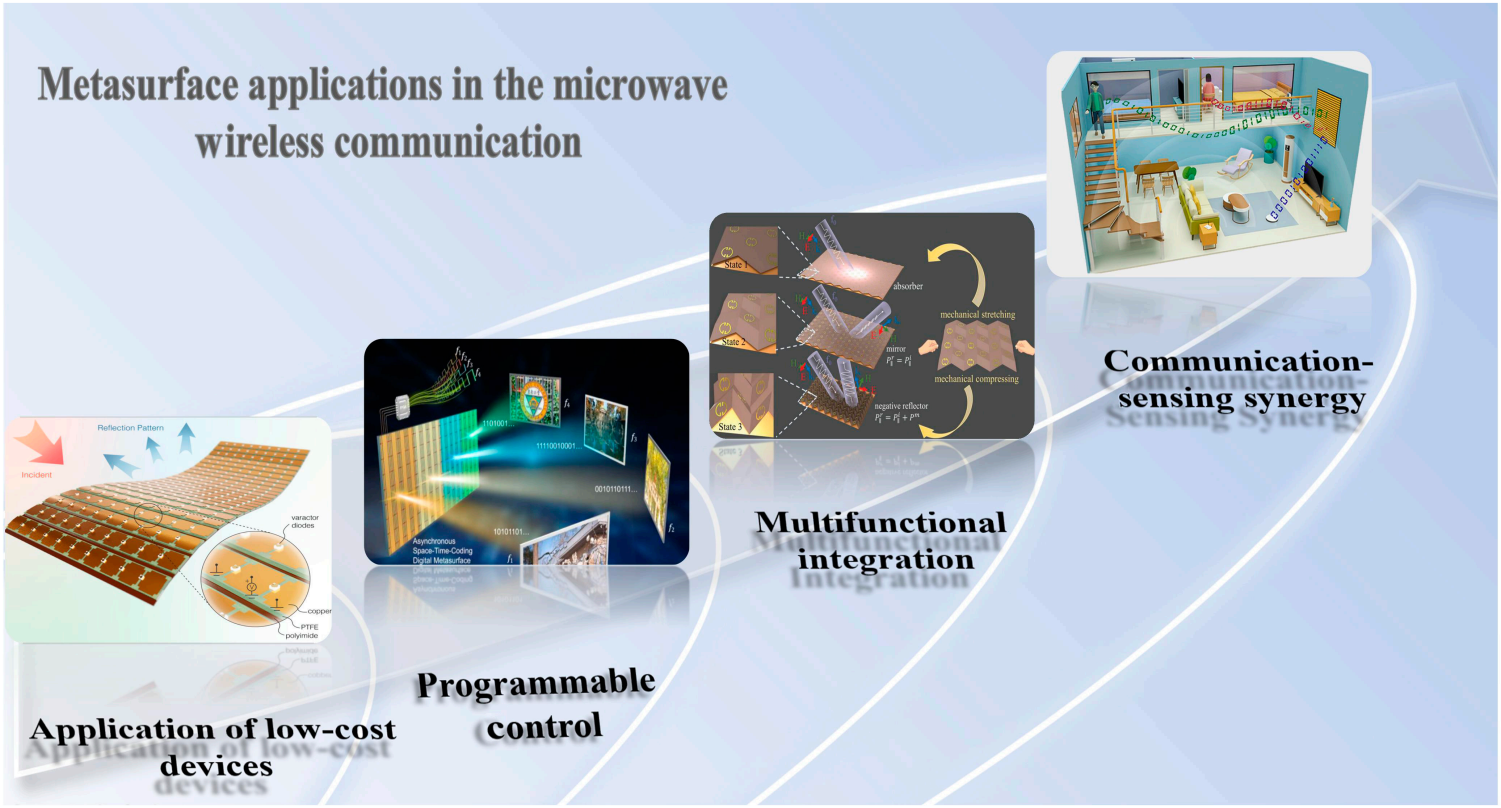
Evolution pathway of metasurface technologies in communication from fabrication feasibility to intelligent integration [64–67].

With the accelerated development of 5G and 6G technologies, modern communication systems are increasingly facing challenges such as spectrum scarcity, complex channel conditions, and energy efficiency bottlenecks [52–55]. Microwave metasurfaces—engineered structures capable of precise electromagnetic manipulation—provide highly flexible and integrated solutions for next-generation communication architectures [56–58]. As shown in Fig. 2, we can summarize the development direction of microwave metasurfaces into 3 stages: manufacturing feasibility, programmable control, and multifunctional integration and communication-sensing fusion. The practical implementation of microwave metasurfaces initially hinges on the development of cost-effective fabrication techniques [59–62]. In recent years, methods such as printed circuit boards, 3D printing, and laser direct writing have enabled the realization of large-area or flexible microwave devices. These technologies have been widely applied in subsystems such as antennas, filters, absorbers, and interference suppression modules, providing essential support for portable, wearable, and environment-embedded communication devices [63].

Building upon this manufacturing foundation, research efforts have increasingly focused on enhancing metasurfaces’ adaptability to complex wireless environments, catalyzing the rapid development of reconfigurable structures [64–67]. Programmable reflecting surfaces (PRS) or intelligent reflecting surfaces (IRS) can dynamically control the phase and beam direction of incident waves. These technologies effectively mitigate multipath interference and support non-line-of-sight transmission, enabling real-time reconfiguration of wireless channels and significantly improving link reliability and energy efficiency—making them foundational to the realization of intelligent communication environments. Advancing beyond reconfigurability, metasurfaces are evolving into multifunctional integrated platforms. Through synergistic structural and material design, a single device can simultaneously achieve beam shaping, polarization conversion, frequency selectivity, and electromagnetic absorption, thereby simplifying system architecture. For instance, dual-band phase-coded metasurfaces have demonstrated the ability to switch between different communication protocols, offering enhanced communication security and environmental adaptability for demanding applications such as unmanned systems and satellite links. Looking ahead to 6G, communication systems are transitioning from connectivity-driven paradigms to integrated communication–sensing–intelligence frameworks. Sensing-enabled metasurface networks, realized through distributed deployment, possess real-time awareness of user location, behavior, and environmental obstructions, enabling dynamic optimization of communication paths. Combined with artificial intelligence (AI) algorithms and programmable structures, future “meta-atom networks” are expected to achieve autonomous spectrum management, multi-user coordination, and collaborative communication.

Thus, it is necessary to thoroughly review the development of metasurfaces and metamaterials to identify the key research directions for the coming future. In this review, we summarize the typical applications of metamaterials and metasurfaces in the THz and microwave frequency bands. Specifically, we focus on the latest developments in the use of such engineered platforms for biosensing in the THz band and their applications in microwave wireless communications. The aim is to highlight the recent advances and application potentials of these technologies in both microwave wireless communication and THz biosensing. As these technologies continue to evolve and improve, they are expected to play an increasingly critical role in future scientific research and industrial applications, bringing additional innovations and value to society.

## Metasurface Applications in the THz Sensing

With the increasing demand for sensitive and selective biological sensing, conventional metallic THz metasurfaces have played a pivotal role in enhancing the detection of minute refractive index perturbations, owing to their mature fabrication processes, flexible structural designs, and strong localized resonance capabilities. However, limitations in selectivity and dynamic tunability still hinder their application in more complex sensing scenarios. To overcome these limitations, electrically tunable graphene has been incorporated into hybrid graphene–metal THz metasurfaces, enabling active control over resonance characteristics and expanding their potential for reconfigurable and multimodal sensing. In recent years, the development of high-specific surface area carbon nanomaterials has led to the integration of CNTs into THz metasurfaces. Their outstanding molecular adsorption capacity, biocompatibility, and surface functionalizability provide a promising route for achieving highly sensitive and selective detection of trace analytes. It is worth emphasizing that with the development of materials science, THz metasurface sensors based on other material systems (dielectrics, new 2D materials, phase change materials, etc.) are also being explored to further improve their performance and meet complex detection requirements.

In this review, we focus on 4 representative material frameworks: (a) metal-based THz metasurface biosensors, (b) graphene–metal composite THz metasurface biosensors, (c) CNT film-based THz metasurface biosensors, and (d) all-dielectric THz metasurface biosensors. In parallel, 3 functional strategies are adopted as complementary perspectives to evaluate sensing performance: label free refractive index sensing, specific molecular recognition through functionalization, and pixelated metasurface-based fingerprint spectral reconstruction. By combining material classification with targeted detection strategies, we systematically review the structural designs, enhancement mechanisms, and application scenarios of each category, and then clarify the evolutionary trends and technical challenges of THz metasurfaces for trace biochemical detection.

### Metal-based THz metasurface biosensors

**Fig. 3.**
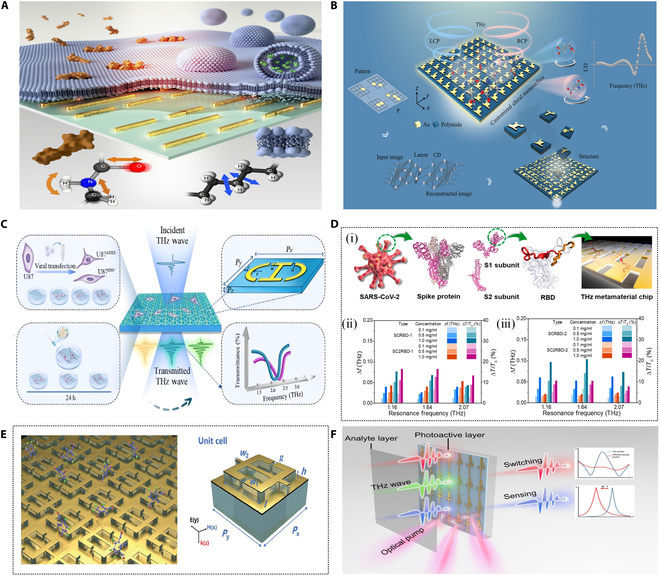
Metallic THz metasurface sensors based on different resonance modes. (A) Schematic diagram of label-free chemically specific metasurface sensors for distinguishing multiple analytes in dynamic lipid membrane processes [68]. (B) Metallic THz chiral metasurfaces for biosensing applications [69]. (C) Schematic diagram of the sensing strategy of the meta-biosensor based on the toroidal dipole mode [71]. (D) Metamaterial sensors composed of nanoslot arrays for detection and discrimination of SARS-CoV-2 and SARS-CoV (i), SCRBD-1 (ii), and SCRBD-2 (iii) using THz quantitative analysis results [72]. (E) Floating bilayer metasurface sensor for highly sensitive detection of thiophanate-methyl. (i) Schematic diagram of metasurface array. (ii) Schematic diagram of unit structure. (iii) Transmission spectrum of metasurface sensor tested in the experiment; inset is an optical microscope image. (iv) THz transmission spectra of metasurface loaded with different concentrations of thiophanate-methyl [78]. (F) Schematic diagram of plasmonic metasurface with ultralow pump threshold, ultrafast switching cycle time, and high sensitivity [79].

Metallic THz metasurfaces are engineered to exhibit unique electromagnetic resonance phenomena that are absent in natural materials, achieved through the precise design of metallic micro- and nanostructures. As shown in Fig. 3A and B, with their flexible design, high sensitivity, and broad application potentials, metal-based THz metasurfaces have become a key focus in the development of THz sensing technologies [68]. For example, a pioneering photon-induced ultrafast programmable THz logic gates was created via pixelated design with hybrid multi-materials and different polarizations on meta device [69]. According to the different resonance modes, they can be classified into spoof localized surface plasmons, toroidal dipole mode, electromagnetically induced transparency (EIT), Fano resonance, and quasi-bound state in continuous media, to name a few. These resonance modes are extremely sensitive to changes in environmental parameters, such as refractive index and absorptivity [70].

In [71], Mu et al. proposed a THz metasurface biosensor, as depicted in Fig. 3C, for detecting and distinguishing different subtypes of glioma cells. The research results show that when a THz beam irradiates the surface of the biosensor, 2 circular currents with opposite spin directions are excited in the 2 open resonant rings of the device, creating a head–tail magnetic field confined within the unit cell. This circular magnetic field induces a polarization-insensitive toroidal dipole resonance at 2.12 THz. When the proposed biosensor is loaded with glioma cells, the transmission spectrum undergoes noticeable shifts in resonance frequency and amplitude, reflecting these differences. To further validate the biosensor, the researchers stained the cytoskeleton of various glioma cell subtypes and analyzed morphological changes for different concentrations. Experimental results demonstrate that the ring dipole resonance-based biosensor holds significant potentials for clinical and intraoperative molecular pathology diagnosis, as well as chemical drug screening.

Beyond cell testing, virus detection represents another critical application area in life sciences, medical research, and clinical diagnostics. While cell studies focus on functions and characteristics, virus research emphasizes their existence and behavior, offering complementary insights into these biological systems. In [72], Lee et al. utilized a metamaterial sensing chip combined with a THz time-domain spectroscopy (THz-TDS) system to investigate the detection of receptor-binding domain (RBD)-derived peptides in severe acute respiratory syndrome coronavirus 2 (SARS-CoV-2) and their sensitive and selective detection in SARS-CoV, as shown in Fig. 3D (i). Using this approach, metamaterial sensor chips with resonance frequencies of 1.16, 1.64, and 2.07 THz were employed to experimentally study the optical responses of SCRBD-1 (AA residue 427–446) and SC2RBD-1 (residue 440–459), as well as SCRBD-2 (residue 447–466) and SC2RBD-2 (residue 460–479), which have similar secondary structures, in the THz range. Figure 2D (ii) and (iii) summarizes the average concentration-dependent shifts in each resonance peak (Δ𝑓) and relative transmission change (Δ𝑇/𝑇_0_) of the metamaterial sensor. A highly linear relationship was observed across all concentrations (*R*^2^ > 0.98). Quantitative analysis revealed a detection limit of approximately 0.1 mg/ml (41.7 μM) for all peptides tested. This study highlights not only the effectiveness of the metamaterial-based THz sensor for detecting biomolecules within the same genus or even at the peptide level but also the significance of this THz-TDS analytical platform for point-of-care, label-free diagnostic applications.

**Fig. 4.**
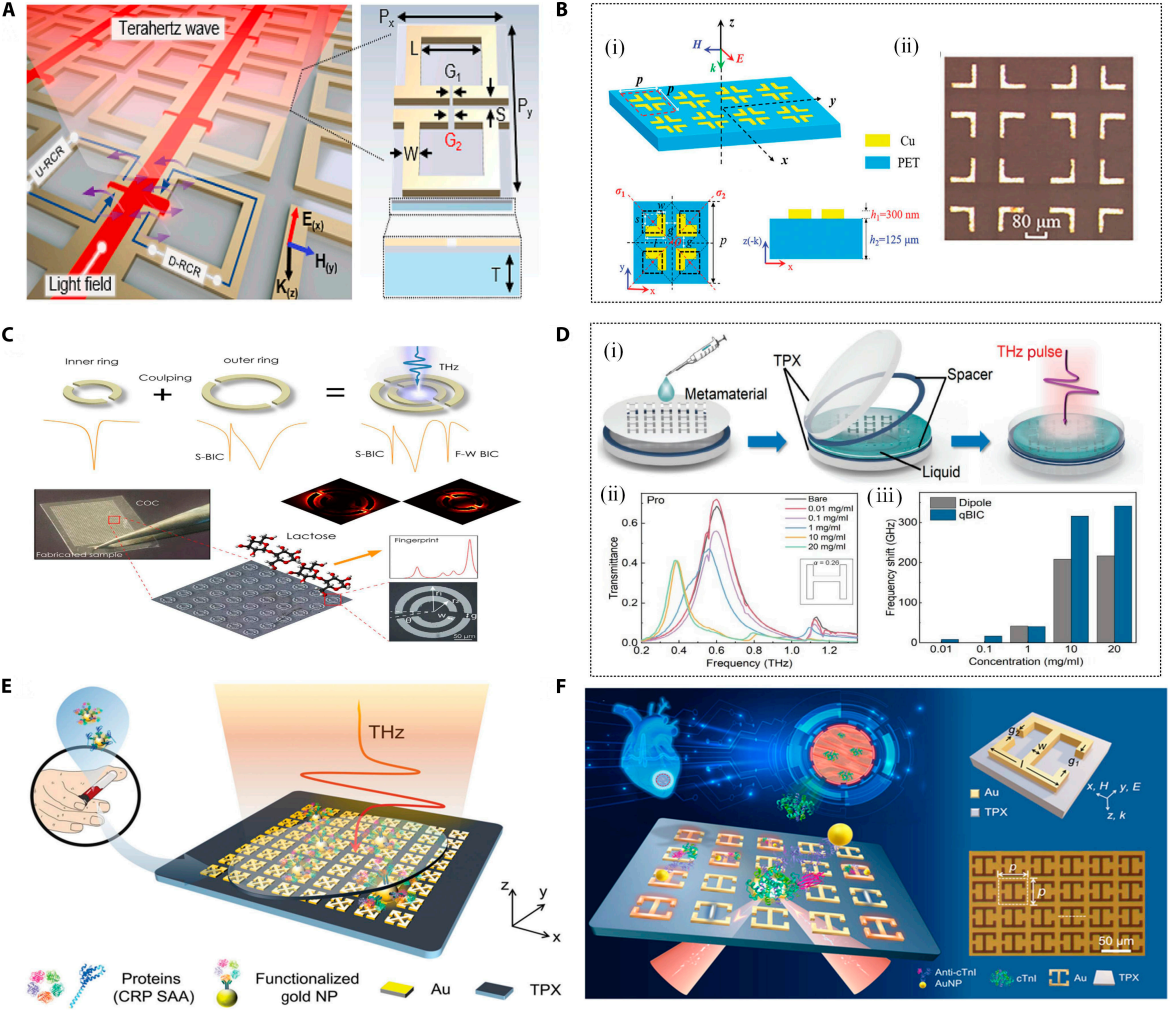
Metasurface sensors based on QBIC modes. (A) QBIC metasurface sensor consisting of 2 ring-chain resonators with different gap widths and geometrical parameters of the lattice [80]. (B) Schematic diagram and micrographs of a tetramer-based metasurface supporting 3-band QBICs, where the black dashed line indicates the position before a specific shift [82]. (C) Schematic diagram of a multi-band BIC metasurface sensor consisting of a double split resonant ring (inner ring and outer ring) and its THz transmission spectrum [83]. (D) (i) Metasurface sensor for alcohol detection. (ii) Transmittance spectra of the sensor at different concentrations of Pro molecules. (iii) Resonance frequency shift diagram of dipole and QBIC [84]. (E) Schematic diagram of an on-chip ultrasensitive THz plasmonic metasensor for distinguishing extremely dilute concentrations of solutions through quasi-BICs [39]. (F) Structural schematic diagram of a THz QBIC metasurface sensor for trace troponin detection based on biofunctionalization [85].

In the field of biosensing, improving the detection sensitivity of THz metasurface sensors significantly enhances their ability to detect subtle changes in molecular configurations on cell surfaces, thereby increasing the efficiency of early disease screening [73–76]. However, environmental noise always influences the accurateness and robustness of matter sensing. Groundbreaking research that a single laser-controlled metasurface generates 2 transmission spectra between picosecond time delays was created to achieve the calibration-free, high precision, and robust sensing [77]. Therefore, in order to reduce the influence of the substrate on the sensitivity of the metasurface sensor. Liu et al. [78] proposed a high-performance THz sensor based on a floating bilayer metasurface utilizing ring dipole resonance, as shown in Fig. 3E. The sensor demonstrated outstanding performance in chlorothalonil detection experiments, achieving a frequency shift sensitivity of 362 GHz RIU^−1^, approximately 2.6 times higher than that of conventional metasurfaces. Furthermore, by optimizing the floating height (with 10 μm identified as optimal), the sensor’s sensitivity could be further enhanced, showcasing remarkable design flexibility. The high-quality factor (*Q*-factor) resonance of the floating bilayer metasurface enables a highly concentrated electric field intensity near the resonator. Both experimental and simulation results demonstrated a pronounced redshift in response to variations in analyte thickness and refractive index, highlighting the sensor’s excellent detection capabilities. In theory, a higher *Q*-factor enables a metasurface to respond more precisely to target frequency changes and enhances its ability to detect tiny signal fluctuations. BIC metasurface structures typically exhibit higher *Q*-factors, making them highly responsive to electromagnetic waves within specific frequency ranges. For THz-band metasurface sensors, the BIC phenomenon allows electromagnetic waves to form bound states within the structure, resulting in significant local field enhancement. As shown in Fig. 3F, Ren et al. [79]. used this concept to construct a quasi-BIC (QBIC) metasurface. This enhancement substantially improves the sensor’s sensitivity to minor changes, such as molecular adsorption or refractive index variations, enabling ultrasensitive detection of low-concentration target substances. In [80], Liu et al. designed a highly sensitive THz biosensor based on QBIC, as shown in Fig. 4A. This sensor leverages asymmetry introduced in a 2-ring chain resonator structure with different gap widths to manipulate the interferometric coupling between electric quadrupoles and magnetic dipoles, thereby exciting a QBIC resonance with an ultrahigh *Q*-factor. Simulation results demonstrate that by breaking the symmetry-protected BIC state, the metasurface achieves QBIC resonances with *Q*-factors as high as 503 in the far field. Experimentally, a low-concentration aqueous solution of homocysteine (Hcy) was chosen as the test subject. Hcy samples of varying concentrations were evenly distributed and randomly attached to the metasurface film via the drop-drying method. The sensor achieved a direct limit of detection (LoD) of 12.5 pmol/μl, at least 40 times higher than the dipole mode’s LoD of 0.5 nmol/μl. This indicates that the QBIC-based metasurface biosensor is capable of detecting trace amounts of Hcy molecules at the pmol level.

In biomedical testing, microfluidic systems are particularly well suited for continuous or dynamic monitoring of biological samples and are easier to integrate with automated equipment [81]. In [82], Ding et al. utilized laser etching technology to achieve low-cost, large-scale manufacturing of THz metasurface sensors, as illustrated in Fig. 4B. The structural unit enables the transformation of symmetry-protected BIC (SP-BIC) into dual-band or triple-band QBIC through collective perturbation, specific displacement, or their combination. Since the tetramer maintains 𝐶4𝑣 symmetry throughout operation, the resulting QBICs remain polarization independent. By integrating the metasurface with a microfluidic channel, Ding et al. developed a QBIC-based THz microfluidic biosensor. This sensor was employed to measure bovine serum albumin solutions at varying concentrations. Experimental results revealed that, for BSA concentrations below 4 mg/ml, both resonance modes of the dual-band sensor exhibited a sensitivity of approximately 12 GHz/(mg/ml), with a detection limit of 0.17 mg/ml. This study not only presents a straightforward strategy for designing metasurfaces supporting multi-band, polarization-independent QBICs but also highlights their potential applications in biosensing, filtration, and laser technologies.

In contrast to previous studies focusing on low *Q*-factor resonant structures or single QBICs, Yang et al. [83] experimentally demonstrated the feasibility of a THz metasurface based on double split-ring resonators for ultrasensitive fingerprint detection of α-lactose, as shown in Fig. 4C. Lactose, a disaccharide composed of d-glucose and d-galactose linked by a β-1,4 glycosidic bond, exhibits multiple characteristic absorption peaks in the THz frequency range. By modulating the asymmetry induced by geometric parameters of the inner and outer rings, this metasurface sensor achieved multiple, freely tunable Friedrich–Wintgen BIC resonances at 0.5, 0.95, and 1.17 THz. These resonances corresponded precisely to the characteristic fingerprints of α-lactose. Dried lactose molecules were evenly distributed on the resonator surface, and light–matter interactions at a lactose concentration of 2.77 nmol/μl were analyzed. Experimental results demonstrated significant absorption enhancement at all lactose absorption peaks. The maximum absorption enhancement, calculated by dividing the metasurface’s maximum absorption by the reference value of the lactose-containing substrate, reached 116.5.

In addition, Lin et al. [84] experimentally demonstrated a tunable QBIC THz metamaterial sensor composed of an H-shaped array, as shown in Fig. 4D. By modulating the asymmetry of the microstructure, the symmetry-protected BIC can be converted into a QBIC with enhanced local field and flexible tunability. Using this tunable property, liquid sensing characterization based on low *Q*-factor and high-intensity resonance under large asymmetry is achieved. Although the aforementioned biosensors based on THz metasurfaces combine subwavelength-scale structural design and electromagnetic response characteristics, breaking through the limitations of traditional optical sensing technologies to some extent and demonstrating unique advantages in biomolecule detection, they still face several limitations and challenges. For instance, conventional metasurface sensors often exhibit similar responses to multiple molecules, lack molecular recognition capabilities, and struggle to differentiate between similar molecules or perform specific detection in complex biological samples. To address these challenges, highly selective interfaces can be constructed on metasurfaces through specific modifications, such as functionalized gold nanoparticles (AuNPs), molecular imprinting polymers, or biological probes (e.g., antibodies and aptamers). As shown in Fig. 4E, a novel metasurface sensor was created by periodically arranged split-ring resonators combined with functional colloidal AuNPs for trace protein detection [39]. This outstanding approach has great potential for precise detection of specific molecules in complex biological environments.

In [85], Wang et al. developed an ultrasensitive THz metasurface sensor by combining functional colloidal AuNPs with photolithography and magnetron sputtering techniques, as illustrated in Fig. 4F. The metasurface design employs a metal open-ring resonator and achieves the transition from a nonradiative BIC to an observable high *Q*-factor QBIC by breaking the structural symmetry along the *y* axis. The QBIC mode is predominantly driven by the magnetic dipole mode, while the low-order mode is sustained by the electric dipole mode. Experimentally, the integration of functionalized AuNPs, modified with specific antibodies, and the QBIC metasurface enabled ultrasensitive and specific detection of cardiac troponin I. The experimental results demonstrated a strong linear relationship between protein concentration and the resonance frequency of the metasurface. The sensor achieved a detection limit as low as 0.5 pg/ml, an order of magnitude lower than existing methods.

**Fig. 5.**
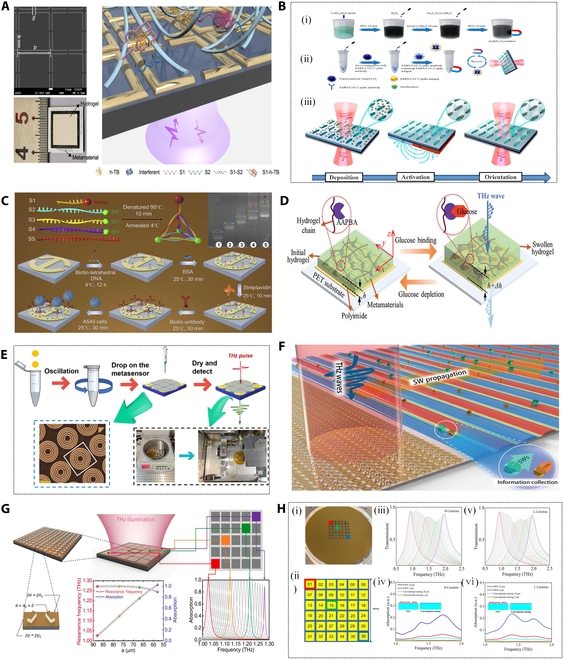
Specific detection of trace biochemical substances based on THz metasurfaces. (A) THz metamaterial SEM images, detection schematics, and physical images that can be used to develop various molecule-specific THz biosensors [86]. (B) Design of a biosensor detection platform based on magnetic particles and metamaterials. Preparation of Au@Fe_3_O_4_ (i) and specific recognition process of SARS-CoV-2 (ii). Migration process to the THz metamaterial unit structure under the action of an external magnetic field (iii) [87]. (C) Schematic diagram of self-assembly of tetrahedral DNA nanostructures that can achieve high specificity tracking detection [88]. (D) (i) Schematic diagram of the AAPBA hydrogel-based THz metamaterial combined with glucose biosensor. (ii) The sensor was used to detect glucose concentration in the serum of 3 healthy people [89]. (E) Detailed experimental protocol of an *N*-order concentric circle metasensor supporting AIT for specific recognition of multiple and mixed chemicals and optical microscopy images of the fabricated metasensor [93]. [F] Schematic diagram of refractive sensing and fingerprint recognition leveraging long-range transmission, strong confinement, and interface sensitivity of surface waves [94]. [G] Schematic diagram and absorption spectrum of a pixelated QBIC metasurface biosensor for identifying and quantifying the vibrational absorption fingerprint features of different biomolecules in the THz band [95]. (H) (i) FSFS based on a THz metasurface for enhanced broadband chiral enantiomer multiplex signals and narrowband molecular AIT-specific detection. (ii) Schematic diagram of the 36-pixel array structure. Normalized THz transmission spectra of d-carnitine (iii) and l-carnitine (v) simulated by FSFS multiplexing; absorbance envelopes of d-carnitine (iv) and l-carnitine (vi) with different thicknesses simulated by conventional sensing and FSFS [96].

Furthermore, Zhou et al. [86] used aptamer hydrogel-modified THz metamaterials to prepare a molecular-specific THz biosensor for highly sensitive detection of changes in the hydrogel hydration state caused by trace amounts of thrombin, as shown in Fig. 5A. The experimental results showed that the optimized THz sensor had good specificity and sensitivity for the detection of actual serum samples, and the detection limit in the human serum matrix was relatively low, at 0.40 pM. This example illustrates how the combination of functionalized AuNPs and THz metasurfaces can significantly enhance both the sensitivity and specificity in the detection of trace biological samples. Such advances highlight the potential of this approach in precision biosensing applications. To further enhance the reusability and flexibility of this metasurface sensor, Bi et al. [87] innovatively integrated magnetic nanoparticles with THz metamaterials, as depicted in Fig. 5B, to achieve highly sensitive detection of the SARS-CoV-2 virus spike protein. Magnetic nanoparticles were employed to selectively bind target molecules and migrate toward the THz metamaterial unit structure under the influence of an applied magnetic field. By precisely controlling the interaction between magnetic nanoparticles and target molecules, this technology accurately identified the location of the SARS-CoV-2 spike protein on the groove surface of the THz resonator. In this approach, functionalized magnetic nanoparticles were synthesized via a hydrothermal method and covalently linked to AuNPs and specific antibodies to enhance antibody binding sites.

Subsequently, an array of elliptical notches was fabricated on a metal substrate through a wet isotropic etching process, forming the metamaterial structure. The efficiency of the proposed sensor was confirmed through comparative analysis of real samples under magnetically controlled and nonmagnetically controlled conditions. Results demonstrated a strong linear relationship between the resonant frequency of the metamaterial sensor and

the concentration of SARS-CoV-2 spike protein in the range of 0.005 to 1,000 ng/ml, with a detection limit of 0.002 ng/ml and a frequency shift of 24 GHz. The sensor also exhibited a stability index of 95%. Further assessments of selectivity, repeatability, and stability confirmed the sensor’s high performance in detecting SARS-CoV-2 spike protein. In addition, Shen et al. [88] further endowed the microfluidic sensor with immune recognition properties by combining streptavidin-functionalized tetrahedral DNA nanostructures and biotinylated antibody-modified metasurface sensors with microfluidic channels, as shown in Fig. 5C.

In [89], Zhang et al. combined THz metamaterials with 3-acrylamidephenylboronic acid (AAPBA)-based hydrogels to propose a novel THz metamaterial biosensor, as shown in Fig. 5D. This sensor is capable of detecting glucose in solution, serum, and sweat with high sensitivity and specificity. The underlying mechanism is that glucose can reversibly react with the AAPBA component, altering the hydrogel’s water content—a subtle change that can be detected by the THz metamaterial sensor. In this study, AAPBA hydrogel was integrated with an aluminum double split-ring resonator array to create a flexible THz metamaterial sensor platform. The sensor demonstrated the ability to detect glucose concentrations ranging from 0.005 to 1,000 ng/ml, with a detection limit as low as 1.64 mg/dl, showcasing excellent sensitivity and stability. Experimental results indicated that as glucose concentration increased, the transmission peak of the sensor decreased, corresponding to the swelling of the hydrogel. The researchers further evaluated the sensor’s accuracy in detecting glucose concentrations in human serum and sweat. The results showed that the sensor effectively enables noninvasive, real-time monitoring, offering new possibilities for diabetes management.

In summary, the application of specifically modified THz metasurfaces and metamaterials for trace biological sample detection has significantly improved the selectivity and sensitivity of THz-based sensing, particularly for detecting specific target molecules. Recently, detection solutions utilizing THz fingerprint spectrum sensing technology have garnered widespread attention due to their unique advantages, including high specificity, noninvasiveness, rapid analysis, and exceptional sensitivity. THz fingerprint spectrum sensing technology leverages the distinctive absorption and transmission characteristics of chemical substances in the THz frequency range, which are directly related to the molecular vibration and rotation modes [90–92]. For example, a pioneering self-similar multi-QBICs with gradually changed resonant frequencies was demonstrated to realize independently regulated simultaneous enhancement for broadband spectral detection [41]. They make it possible to precisely distinguish compounds with similar structures, accurately identify chemical substances, and conduct noninvasive detection capabilities.

In [93], Huang et al. developed an *N*-order concentric circle metasensor supporting absorption-induced transparency (AIT), as illustrated in Fig. 5E. Unlike most existing studies that are limited to AIT detection of single biochemical samples, this metasensor demonstrates the capability to specifically identify multiple and mixed substances. The proposed *N*-order concentric ring metasensor achieves selective detection by tuning its structural parameters to match the unique imaginary fingerprint spectra of specific biochemical substances. In their experiment, the authors designed and fabricated a 5-order concentric ring structure capable of simultaneously detecting 4 distinct chemicals: α-lactose, benzoic acid, vitamin B2, and 2,5-dichloroaniline. Experimental results revealed that the metasensor could specifically detect these substances and distinguish their mixtures. The respective detection limits were 8.61, 6.96, 7.54, and 8.35 mg/ml, with sensitivities of 0.00211, 0.00208, 0.00211, and 0.00219 (unit: 1/mg/ml). By analyzing the resonance frequency shifts caused by the real part of the dielectric constant, the metasensor successfully achieved specific fingerprint detection of multiple biochemical substances. However, challenges remain in trace substance detection due to resonance shifts introduced by nondispersive components of the analyte and manufacturing imperfections in the metasurface. These factors can cause the metasurface’s transmission peak to deviate from the THz resonance absorption spectra of the bare analyte. Recently, multiplexed sensing arrays based on THz metasurfaces have been employed for molecular fingerprint recognition and the sensing of chiral enantiomers and isomers, showcasing their potential for high-precision and multi-target biochemical analysis. As shown in Fig. 5F, Zhang et al. [94] pioneered a breakthrough method of quantitative refractive sensing and qualitative fingerprint recognition was created by using long-range THz surface waves excited by metasurface. Yang et al. [95] achieved simultaneous resolution of multiple fingerprint information in a continuous spectrum by collecting broadband information of surface waves and constructing a pixelated THz metasurface sensor strategy, which can also realize the identification and detection of trace substances on the THz metasurface, as shown in Fig. 5G.

In [96], Lyu et al. developed a THz frequency-selective fingerprint sensor (FSFS), as shown in Fig. 5H, designed for enhanced broadband chiral enantiomer multiplexing signals and narrowband molecular AIT detection. Such FSFS, based on a polarization-independent reconstructed metasurface array composed of symmetrical cross-slot resonators, achieves enhanced absorption of trace chiral carnitine over an ultrawide frequency range of 0.95 to 2.0 THz, as well as improved detection of the narrowband fingerprint of α-lactose. Experimental results demonstrate that the FSFS effectively detects the broadband absorption lines of chiral carnitine (d-carnitine and l-carnitine) and the narrowband fingerprint of α-lactose. By adjusting the geometric parameters of the metasurface resonator, the FSFS enhances absorption of trace chiral carnitine within the ultrawide frequency band, achieving an absorption enhancement factor of approximately 7.3. Notably, the FSFS also exhibits the AIT effect in adjacent pixel groups, with a maximum enhancement factor of 7. For α-lactose, the FSFS demonstrates robust AIT performance with an absorption enhancement factor of about 7. The design and experimental validation of the FSFS highlight its ability to significantly enhance the detection of THz molecular fingerprints through AIT and multiplexing technologies.

In general, metasurface biosensors based on metallic materials have garnered significant attention due to their unique optical properties and biocompatibility. These sensors primarily achieve high-sensitivity detection of target molecules through 3 main mechanisms: refractive index sensing, specific surface modification, and fingerprint spectrum sensing. Refractive index sensing leverages changes in refractive index induced by interactions between biomolecules and the sensor surface [97,98]. This approach is straightforward, rapid, and suitable for real-time biomolecule monitoring. However, it may be influenced by environmental conditions and the physical state of the sample, requiring precise control and calibration to maintain accuracy. Specific surface modification involves introducing biorecognition molecules such as antibodies or nucleic acid probes onto the metasurface. This enhances the sensor’s selectivity and broadens its applicability for detecting specific biomolecules. Fingerprint spectrum sensing exploits the unique vibrational and rotational modes of biomolecules in the THz band. By utilizing metasurface heterogeneous arrays, the interaction between THz waves and biomolecules is enhanced, enabling the detection and reproduction of these molecular fingerprint signals. This approach provides molecular-level insights, facilitating the identification of structurally similar molecules, including isomers. It holds significant promise in biomarker detection, disease diagnosis, and environmental monitoring. The development of these techniques has advanced biosensor technology, offering powerful tools for biomedical research, clinical diagnostics, and environmental analysis. However, traditional THz metasurface sensors, typically based on metals or semiconductors, face limitations in the THz band, particularly in achieving high sensitivity, tunability, and multifunctional integration. In contrast, carbon-based materials have emerged as promising alternatives due to their unique physical and chemical properties. These materials demonstrate significant advantages in THz sensor applications, especially in the field of biosensing, and present new opportunities for overcoming the challenges faced by traditional metasurface sensors.

### Graphene–metal composite THz metasurface biosensors

**Fig. 6.**
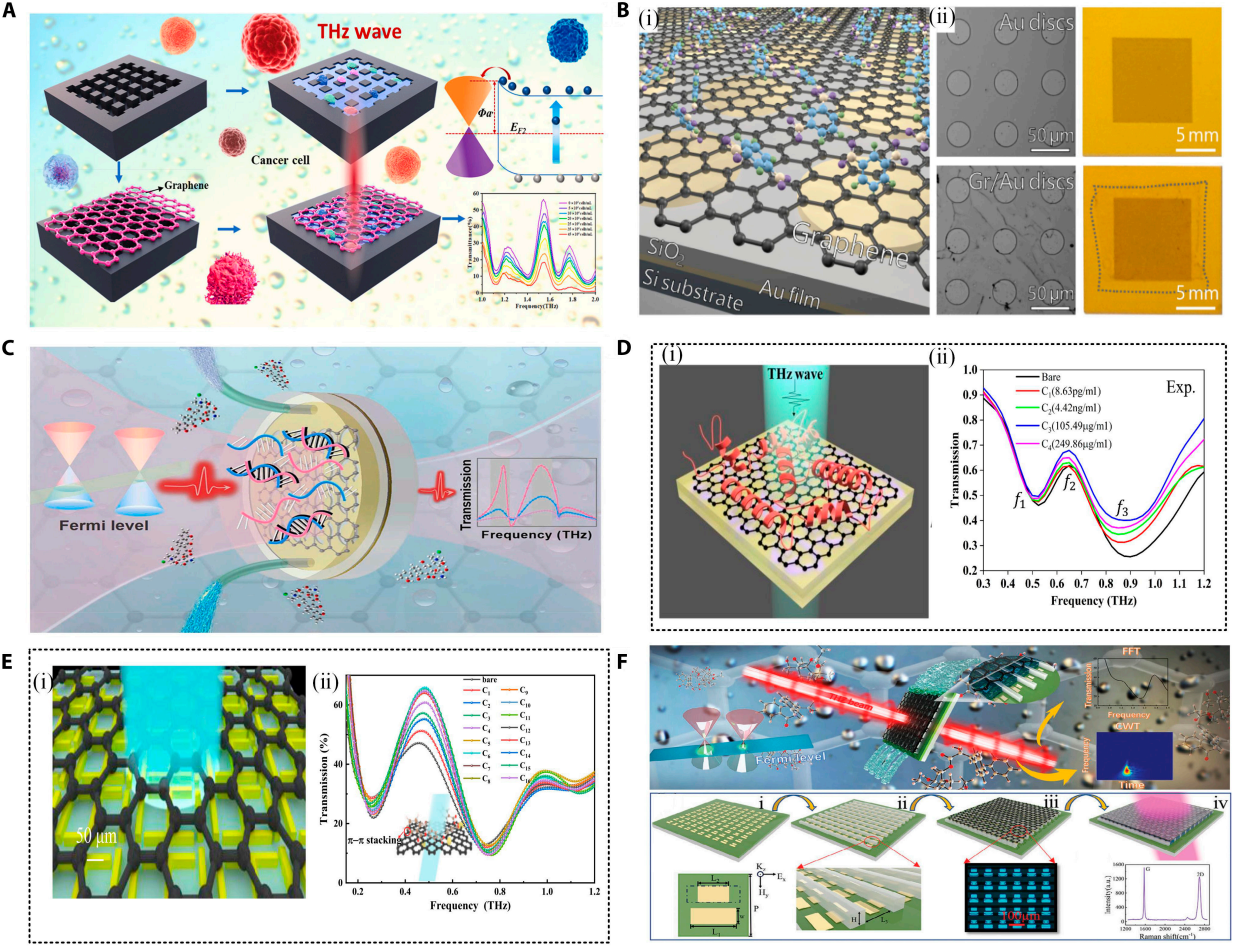
Graphene-assisted THz metasurface sensors for trace substance detection. (A) Schematic illustration of the fabrication process of 2 biosensors for monitoring the concentration of cancer cells [106]. (B) (i) Schematic diagram of graphene-enhanced THz metasurface label-free sensor. (ii) Microscopic images of the fabricated graphene-enhanced high-frequency THz metasurface sensor before and after the transfer of graphene [107]. (C) The CaSR-graphene-based THz microfluidics platform can realize simple, label-free, real-time, and specific biosensing in the THz region [108]. (D) Graphene THz biosensor supporting EIT resonance (i), unit structure of the metasurface, and (ii) graphene composite for the detection of different concentrations of OVA [109]. (E) (i) THz sensor integrating graphene layer, aluminum metal layer, and polyimide substrate for ultrasensitive detection and differentiation of sodium benzoate and potassium sorbate. (ii) Transmission curve of the metasurface sensor as a function of sodium benzoate concentration [110]. (F) Schematic diagram of a sensor based on the combination of QBIC metasurface and graphene [113].

The electronic structure of graphene underpins its outstanding performance in the THz band. As a result, THz metasurface biosensors based on graphene materials leverage its unique physical and chemical properties, combining them with THz biosensing technology to offer a range of significant advantages [99–102]. As a 2D material, graphene boasts high electron mobility, enabling an exceptionally sensitive response in the THz band. Its linear dispersion relation allows graphene to achieve a broad spectral response, which is essential for the development of high-performance and multifunctional THz metasurfaces. In biosensing applications, when target biomolecules adhere to the graphene surface, the conductivity and surface charge distribution of graphene are altered, resulting in a significant shift in the THz response of the sensor. Moreover, graphene’s tunability is another key factor driving its application in THz metasurfaces. The electrical properties of graphene can be regulated through external electric fields, chemical doping, or thermal manipulation. For example, applying an external electric field can adjust the Fermi level of graphene, thereby tuning its optical and conductive properties [103–105]. This enables to dynamically adjust the response characteristics of graphene-based THz metasurface sensors under varying working conditions, making them highly effective for detecting different target molecules. Additionally, the surface of graphene can be chemically modified or functionalized to improve its selectivity and affinity, further enhancing the sensor’s ability to recognize specific biomolecules. As shown in Fig. 6A to C, some high-energy graphene–metasurface composite trace substance sensors have attracted extensive research by researchers [106–108].

In [109], Xu et al. designed and verified a graphene-assisted EIT-like THz metamaterial sensor for ultrasensitive detection of ovalbumin (OVA), as shown in Fig. 6D. By combining graphene, polyimide, and EIT-like metamaterials, the metasensor exhibited a clear transparent window at 0.746 THz and achieved an ultralow detection limit of 8.63 pg/ml. The experimental results demonstrated that for an increase in OVA concentration, the Fermi level of graphene shifts toward the conduction band, causing a decrease in graphene conductivity and an increase in sensor transmittance. As the OVA concentration continues to rise, the Fermi level crosses the Dirac point, leading to an increase in conductivity and a corresponding decrease in sensor transmission amplitude. Additionally, the authors characterized the sensor’s sensitivity to OVA through phase changes, observing that the phase value increased with OVA concentration, reached a maximum at a specific concentration, and then decreased. By comparing the experimental group (with graphene) to the control group (without graphene), the critical role of graphene in the graphene-assisted electromagnetically induced transparency metamaterial (GELM) was confirmed.

As depicted in Fig. 6E, Wu et al. [110] developed a graphene-based metasurface sensor that used quasi-electrode resonance technology to ultrasensitively detect and differentiate 2 common food preservatives, sodium benzoate and potassium sorbate, in the THz region. The conductivity of the graphene layer can be tuned through the preservative-induced electrostatic doping effect, thereby altering the sensitivity of the sensor and enabling it to detect targets at the fM level. In the experiment, the sensor showed higher sensitivity to sodium benzoate, which contains a benzene ring structure. This is due to the π–π stacking effect between sodium benzoate and graphene, which enhanced the sensing effect. By analyzing the measured signals through continuous wavelet transform, the authors constructed a 2D wavelet coefficient intensity map, providing a new method for distinguishing and determining the concentrations of the 2 preservatives. This An–graphene–Ms-based sensor not only quickly detects and distinguishes low concentrations of sodium benzoate and potassium sorbate but also achieves detection limits of 0.12 fg/ml and 0.23 pg/ml, respectively.

Similar to traditional metal-based metasurface sensors, the QBIC approach captures and confines electromagnetic energy within specific optical structures, leading to a significant enhancement of the local light field intensity within the resonant cavity [111,112]. This energy localization can substantially improve sensing performance, making QBIC-based sensors more sensitive to weak changes. Therefore, Huang et al. [113] developed a THz liquid biosensor based on a graphene metasurface and used the concept of QBIC to achieve ultrasensitive detection of various liquids, such as ethanol and *N*-methylpyrrolidone (NMP), as shown in Fig. 6F. By introducing structural asymmetry to excite QBIC resonance and using direct squeezing technology to place the liquid in the field-enhanced region of the metasurface–graphene structure, the interaction between the metasurface and the analyte was enhanced. The minimum detection concentration of the sensor reached 0.21 pg/ml. The sensing accuracy was improved by continuous wavelet transform, and excellent linear fitting results related to the solution concentration changes were obtained, providing a new method for quickly distinguishing solutions of different concentrations. This biosensor, based on the combination of QBIC metasurface and graphene (MGQ), demonstrated excellent sensing capabilities in the THz region under real-time and label-free conditions and was able to quickly distinguish solutions of different concentrations by constructing a standard wavelet coefficient intensity card.

**Fig. 7.**
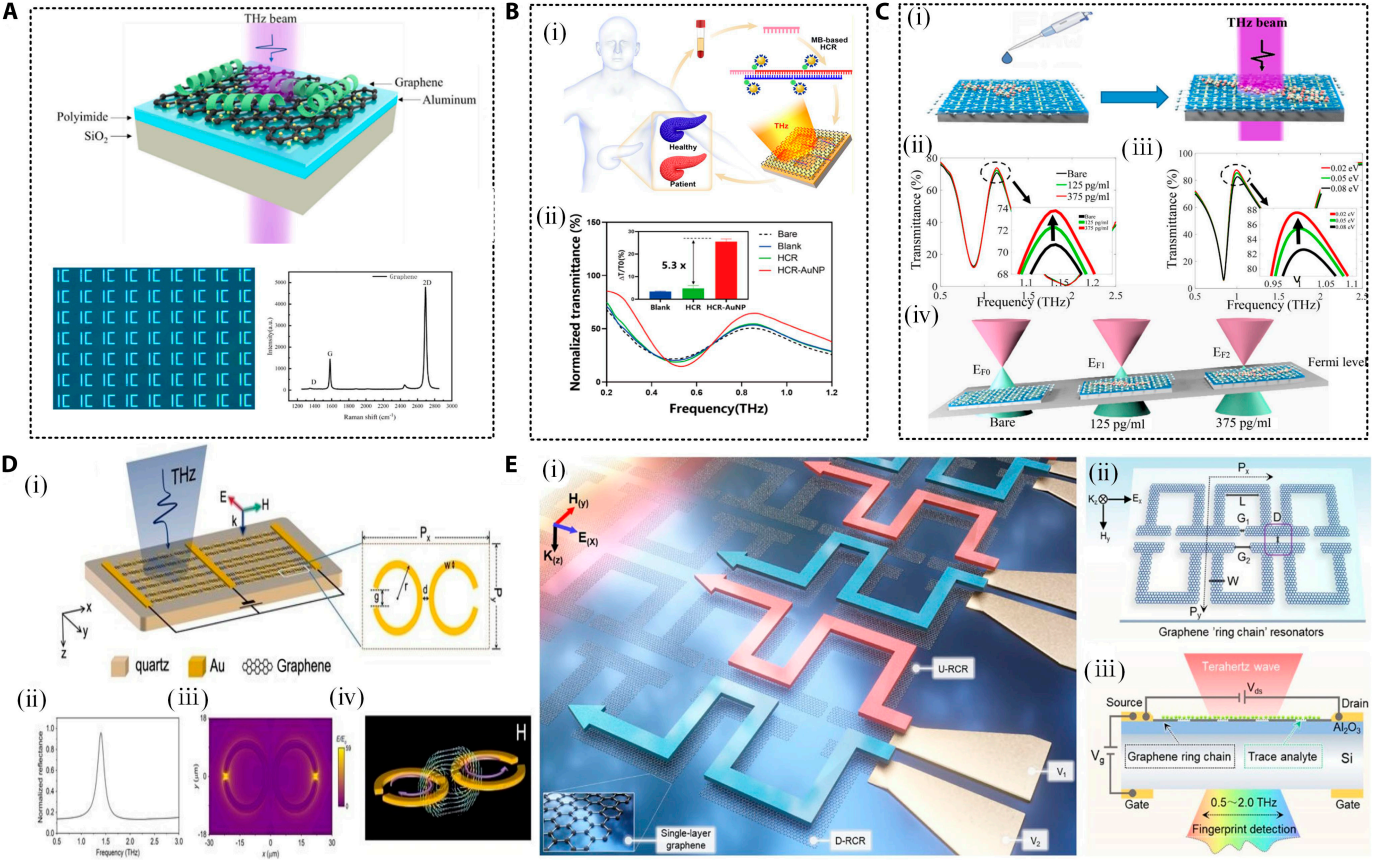
Composite THz metasurface resonance enhancement and specific identification based on nano-doped graphene film. (A) THz metasurface biosensor based on graphene and AuNP integration and its micrograph and Raman shift diagram [114]. (B) Biosensor based on a metal–graphene hybrid THz metasurface for highly sensitive detection of ctDNA. (i) Schematic diagram of the specific detection principle of the metasurface sensor. (ii) SEM images of graphene-coated mass spectrometers with and without HCR-AuNPs. (iii) THz transmission spectra of graphene metasurface, blank control, pure HCR product, and HCR-AuNPs on graphene metasurface. (iv) The metasurface sensor was used to detect normalized transmission spectra of 12 pancreatic cancer patients and 12 healthy subjects [115]. (C) Graphene-integrated toroidal split-ring resonator (TSRR) THz meta-sensor for detecting protein at trace levels based on the Dirac property of CVD graphene [118]. (D) Schematic diagram of enhanced absorption fingerprint spectroscopy detection based on THz pixelated frequency-agile metasurface (i) and normalized reflectance spectrum of symmetric C-ring metasurface sensor (ii). (iii) Enhanced field distribution of metasurface device and clear in-plane toroidal dipole excitation induced by anti-phase magnetic field (iv) [119]. (E) (i) Proposed design concept of reconfigurable graphene metasurface. Second-order absorption fingerprint envelope, natural absorption spectra, and calculated signal enhancement factors of trace l-carnitine (ii) and d-l-carnitine (iii) molecules [120].

In summary, the MGQ biosensor based on the QBIC metasurface provides an effective platform for biosensing in liquid environments, enabling real-time, label-free molecular detection, and multi-dimensional ultrasensitive sensing by detecting changes in resonance intensity and the maximum wavelet coefficient. The work achieved enhanced sensing detection characteristics, such as extremely low detection limits, by combining graphene with metasurface sensors. However, the biological samples detected in the experiment were processed single substances, while real-world applications involve complex biological samples containing multiple substances. Therefore, further research is needed to improve the reliability of detection and the sensor’s selective recognition ability. To enhance the performance of the sensor, AuNPs can be doped into graphene, significantly improving its light absorption and scattering properties. This is of great significance for the development of high-performance optoelectronic devices and sensors. Liang et al. [114] developed a THz metasurface biosensor based on the integration of graphene and AuNPs, as shown in Fig. 7A, aiming to substantially improve the sensitivity of biological sample detection. A AuNP solution was dropped onto the graphene surface and heated and dried to obtain a graphene film integrated with AuNPs. The experimental results show that compared to the sensor integrating only graphene, the sensor integrating both graphene and AuNPs exhibits more obvious amplitude changes when the aspartic acid concentration changes, especially in low concentration amino acid solutions. AuNPs can generate charge accumulation and induce local electric field enhancement due to the tip effect and localized SPR phenomenon, thereby enhancing the interaction between THz waves and analytes. This sensor integrating graphene and AuNPs achieved a detection limit of 10.48 fg/ml when detecting aspartic acid solution.

Based on this mechanism, Luo et al. [115] developed a biosensor based on a metal–graphene hybrid THz metasurface for high-sensitivity detection of circulating tumor DNA (ctDNA), as shown in Fig. 7B. This sensor cleverly combines a dual signal amplification strategy, as illustrated in Fig. 7B (ii). On one hand, AuNPs are used to enhance the charge injection into graphene, significantly altering the transmission characteristics of THz radiation (with signal enhancement by 2 orders of magnitude compared to the configuration without graphene). On the other hand, chemical amplification is achieved through the metasurface and magnetic bead-based hybridization chain reaction (HCR). The introduction of HCR not only amplifies the signal but also ensures that only the target ctDNA can be triggered via a specific DNA hybridization reaction. This signal amplification enables highly specific recognition of target molecules. The design allows the sensor to detect ctDNA in plasma samples from pancreatic cancer patients at the sub-femtomolar level and to distinguish single-nucleotide mismatches, demonstrating extremely high specificity. The results show that the platform can detect targeted ctDNA sequences with a LoD of 0.22 fM, exhibiting good specificity and the ability to distinguish other subtype mutations. It can stably and reliably differentiate pancreatic cancer patients from healthy individuals, showing good consistency with quantitative real-time polymerase chain reaction (PCR) results.

Metamaterial/metasurface fingerprint spectrum sensing detection solutions based on graphene materials have also received widespread attention recently [116,117]. This fingerprint spectrum sensing technology has made significant advances, particularly in its ability to leverage the dynamic adjustment of pixelated frequencies on metasurfaces to achieve broadband spectral detection. By precisely regulating the Fermi level of graphene to linearly adjust the resonance frequency, a wide spectral range can be covered, enabling high-sensitivity and high-resolution molecular fingerprint spectral identification. Additionally, the design allows for the establishment of a one-to-many mapping between spatial and spectral information, which significantly reduces the size of the sensor. This provides a highly efficient, multifunctional miniaturization solution for biochemical analysis and component identification.

The core of this sensing detection scheme lies in the design and tuning capabilities of the graphene metasurface. By adjusting the Fermi level of graphene through voltage, precise control of the resonant frequency in the THz band is achieved. As shown in Fig. 7C, Wang et al. [118] designed and demonstrated a graphene-integrated THz metasensor based on toroidal dipole resonance, leveraging the intrinsic characteristics of chemical vapor deposition (CVD) graphene with its Fermi level slightly deviated from the Dirac point and its pronounced sensitivity to external perturbations. The adsorption of protein molecules induces Fermi level shifts in graphene, thereby modulating its conductivity and resonant behavior, which highlights its inherent electro-tunability. As a result, label-free detection of Midkine at concentrations as low as 125 pg/ml was achieved. Furthermore, the incorporation of carcinoembryonic antigen (CEA) antibody-functionalized AuNPs exploited localized SPR to enhance local electric fields and charge accumulation near the graphene surface, significantly boosting the resonance strength and detection sensitivity. This strategy enabled the ultrasensitive detection of CEA down to 10 pg/ml, demonstrating the strong potential of graphene–AuNP hybrid enhancement mechanisms for THz-based trace protein biosensing.

In [119], Sun et al. successfully developed a broadband THz metasurface sensor based on a pixelated frequency-agile metasurface, achieving high-sensitivity detection and resolution of molecular fingerprint spectra, as shown in Fig. 7D. By combining a symmetric metal C-shaped resonator with a functionalized graphene microstrip, the sensor achieves linear regulation of the resonant frequency in the range of 1.82 to 2.27 THz and exhibits a high spectral resolution of less than 20 GHz. The study found that by changing the Fermi level of graphene, the resonant frequency could be precisely controlled, achieving a frequency change rate of 120 GHz/0.1 eV, providing powerful tuning capabilities for broadband THz spectroscopy detection. This metasurface sensor innovatively links specific metasurface pixels to multiple characteristic absorption spectra, establishing a one-to-many mapping between spatial and spectral information, significantly reducing the actual size of the sensor. Experimental results show that the sensor has a highly sensitive reading capability for the target multiple enhanced absorption spectra of glucose molecules across a broadband region, with the absorption enhancement improving by nearly 5 times. Additionally, by utilizing the natural reconfigurable properties of the metasurface, dynamic reconstruction and slow light modulation of the EIT resonance were achieved through an asynchronous voltage regulation scheme, demonstrating tuning capabilities within an ultrawideband range and modulation speeds at the GHz level. The metasurface not only exhibited excellent performance in fingerprint enhancement and recognition of chiral molecules but also proved its universality in detecting disease markers such as γ-aminobutyric acid molecules. Liu et al. [120] proposed an ultrawideband THz fingerprint-enhanced sensing technology based on a single-pixel reconfigurable graphene metasurface, as shown in Fig. 7E, which achieved ultrasensitive detection of trace analytes such as chiral optical isomers with a detection limit of 0.64 μg/mm^2^. Through synchronous voltage adjustment, the metasurface significantly enhanced the fingerprint signal across an ultrawide bandwidth range of about 1.5 THz, with an enhancement effect reaching 17.4 dB. This enhancement is attributed to the EIT effect and the ideal light field overlap between the graphene monolayer and the ultrathin analyte. A universal fingerprint spectrum inversion model was also developed in the study, effectively addressing the absorption envelope distortion caused by the nonlinear enhancement mechanism of the graphene metasurface in fingerprint retrieval. The model restored the fingerprint signal of the analyte to the standard fingerprint line shape with a correlation coefficient (*R*^2^) of 0.99. Compared to traditional THz metasurface trace biochemical substance sensing and detection technology, this single-pixel graphene metasurface design shows significant advantages in many aspects, including chiral molecular fingerprint enhancement, tuning bandwidth, resonance modulation convenience, system simplification, and chip integration. This innovative approach provides a new technology platform for THz fingerprint sensing, active spatial light modulators, slow light devices, and dynamic imaging devices.

### CNT film-based THz metasurface biosensors

Due to their unique 1D nanostructure, CNTs exhibit exceptionally high electrical conductivity and electron mobility, enabling strong light–matter interactions in the THz frequency range, making them ideal for high-sensitivity sensors. In applications such as biomolecular detection and environmental monitoring, the high specific surface area of CNTs provides more active sites, facilitating efficient interactions with biomolecules or chemical substances [121–125] and further enhancing the ability of THz metasurface sensors based on CNT thin-film materials to precisely respond to subtle changes in analytes, particularly in the detection of low-concentration substances. Similar to graphene, the electrical properties of CNT thin films can be optimized not only by adjusting the alignment, density, and functionalization of the nanotubes but also through external electric field modulation, greatly improving the tunability of the sensors. While graphene preparation methods are well established, challenges persist in achieving large-area uniformity and controllable functionalization. Additionally, graphene production is relatively costly, and its performance may degrade under certain environmental conditions. In contrast, CNT films demonstrate significant flexibility in fabrication methods. Techniques such as CVD, solution-based self-assembly, spin coating, and vacuum filtration enable the production of large-area, high-quality films with precise control over nanotube alignment and density, which is critical for optimizing sensor performance.

**Fig. 8.**
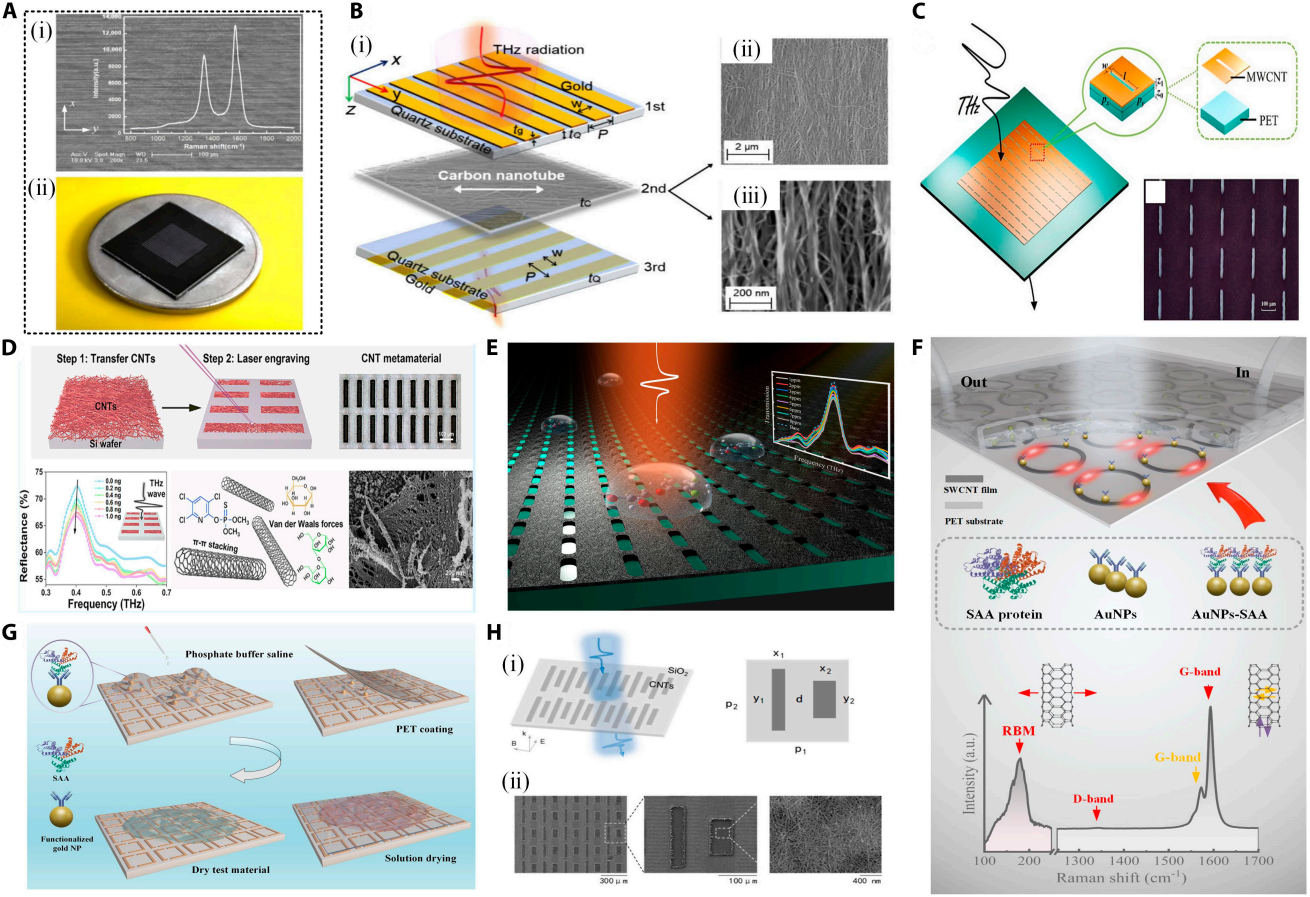
THz metasurface sensor based on CNT film. (A) SEM image, Raman spectrum of anisotropic CNT film, and optical photo of metasurface sensor [126]. (B) (i) Schematic diagram of CNT-integrated metamaterial for orthogonal polarization control. SEM images of the CNT with different magnifications: 2 μm (ii) and 200 nm (iii) [127]. (C) THz metasurface sensor based on MWCNT film [236]. (D) An ultrahigh-sensitivity molecular sensor based on CNT THz metamaterials is composed of CNT cut-wire array on a Si substrate prepared by a novel 2-step method [128]. (E) Image of THz metasurface sensor based on SWCNT film [40]. (F) SWCNT THz metasurface for specific detection of trace amounts of liquid biological samples [237]. (G) Experimental evaluation of SWCNT THz metasurface sensors for detecting ultralow concentration proteins [131]. (H) Schematic diagram of the interaction between SWCNT-based metasurface (i) and THz waves and SEM image of SWCNTs after cysteine coupling (ii) [132].

As a novel nanomaterial, CNT films not only provide high sensitivity, excellent mechanical and thermal stability, good tunability, and flexible preparation processes for THz metasurface sensors but also maintain superior performance under low power consumption and complex environmental conditions. Additionally, CNT materials exhibit outstanding mechanical strength and toughness, with tensile strength and toughness far surpassing many traditional metallic materials. This makes them suitable for applications in flexible electronic devices and wearable sensors, where they can withstand significant mechanical stress. THz metasurface sensors based on CNT thin films exhibit numerous advantages in fields such as biosensing, environmental monitoring, and medical diagnostics. These advantages include high sensitivity, structural tunability, mechanical and thermal stability, low power consumption, and flexible fabrication processes. For example, Wang et al. [126] designed and experimentally fabricated a THz plasmonic metasurface sensor (PMS) based on anisotropic multi-walled CNT (MWCNT) films for pesticide residue detection, as shown in Fig. 8A. The sensor effectively exploits the unique properties of CNTs, particularly their anisotropy, to achieve high-sensitivity detection of pesticide residues, such as 2,4-dichlorophenoxyacetic acid (2,4-D) and chlorpyrifos solutions, in the THz frequency range. The anisotropic nature of CNTs—manifesting as distinct physical and chemical properties along their axial and radial directions—allows for exceptional electrical conductivity and optical performance along the axial direction, which are crucial for facilitating SPR effects in the THz regime. In the experiments, prominent resonant transmission peaks were observed at 0.87 and 1.62 THz. These peaks were successfully interpreted using the Fano model, confirming that the enhanced transmission stems from coupling interactions between surface plasmons (resonant states) and localized surface plasmons (nonresonant states). The experimental dielectric function of the CNT films was also fitted using the Maxwell–Garnett effective medium model, achieving strong consistency with experimental data. This study further validated the feasibility of SPR at the CNT/silicon interface. The PMS sensor demonstrated exceptional sensitivity to pesticide residues at different concentration gradients, achieving a minimum detectable mass of 10 ng. The sensitivities for 2,4-D and chlorpyrifos solutions were 1.38 × 10^−2^/parts per million (ppm) and 2.0 × 10^−3^/ppm, respectively, with a strong linear correlation between transmission amplitude and pesticide concentration. The results indicated acceptable reliability and stability. Interestingly, Xu et al. [127] combined anisotropic CNT films with metal-based THz metamaterials to obtain a THz orthogonal polarization control based on a composite metamaterial (CSMG) sandwiched between a CNT layer and 2 metal grating layers. Since the number of CNT film layers and CNT orientation can achieve highly sensitive polarization conversion of CSMG, this work provides a new option for THz polarization control using nanomaterials with optical microstructures.

Notably, vacuum filtration is widely recognized as a simple, efficient, controllable, and environmentally friendly method for preparing single-walled CNT (SWCNT) films [128,129]. This method enables direct deposition of CNTs onto a variety of substrates, including but not limited to glass, plastics, metals, and semiconductors, significantly enhancing application flexibility. To further explore the performance of this metasurface sensor, its sensing and detection capabilities on different substrates warrant further investigation. As shown in Fig. 8D, Wang et al. [130] prepared an ultrahigh-sensitivity molecular sensor based on CNT THz metamaterials for highly sensitive detection of glucose, lactose, and chlorpyrifos-methyl molecules by vacuum filtration and laser etching. The experimental results showed that the detection limit of this metamaterial sensor was 2 orders of magnitude higher than that of metal metamaterials, and its detection sensitivity was comparable to that of THz graphene metamaterial sensors.

The research team designed a novel THz metasurface sensor based on SWCNT films, as shown in Fig. 8E [40]. For the metasurface construction, the researchers fabricated an isotropic SWCNT film with a thickness of approximately 1 μm on a polyethylene terephthalate (PET) substrate using a vacuum filtration method. Subwavelength array structures with specific periodicity were then etched onto the film surface through laser processing, forming a meticulously designed metasurface. This precise structural design enables the metasurface to generate a significantly enhanced localized electromagnetic field in the THz regime, which is crucial for improving the sensor’s sensitivity. To evaluate its sensing performance, different concentrations of 2,4-D solution were dropped onto the metasurface sensor. The sensor exhibited a detection sensitivity of 2.1 × 10^−2^/ppm and a minimum detectable mass of 10 ng for 2,4-D solutions. These results not only demonstrate the high sensitivity and low detection limit of the SWCNT metasurface sensor in detecting trace chemical substances but also highlight its potential for practical applications.

In addition, SWCNT-based THz metasurface sensors demonstrate significant advances in antigen–antibody specificity modifications, attributed to the excellent biocompatibility of SWCNTs, which allows direct conjugation with biomolecules, such as antibodies, without compromising their biological activity—a critical factor for maintaining antibody functionality and specificity.

In [131], Zhang et al. utilized vacuum filtration and direct laser etching techniques to fabricate periodic asymmetric split-ring resonator (ASR) units on SWCNT films, constructing a modified SWCNT-based THz metasurface sensor for femtomolar-level protein detection, as shown in Fig. 8G. Addressing the challenges of high ohmic losses and low detection sensitivity in conventional THz metasurface sensors for trace biomolecule detection, the study employed sulfuric acid modification of SWCNT films and introduced functionalized AuNPs. This approach significantly reduced the nonresonant losses of the films and enabled specific detection of trace serum amyloid A (SAA). Experimental results revealed a remarkable detection sensitivity of 37.5 GHz/fM for SAA proteins, with a minimum detection limit of 0.1 fM. Compared to traditional metallic or dielectric metasurface sensors, this represents a performance enhancement of an order of magnitude. The SWCNT films were fabricated using the vacuum filtration method to ensure uniformity and quality, and their thickness, surface morphology, and crystalline structure were extensively analyzed using characterization techniques such as scanning electron microscopy (SEM) and Raman spectroscopy. The incorporation of functionalized colloidal AuNPs enabled the sensor to specifically capture trace SAA proteins, achieving high sensitivity detection at femtomolar levels.

Many pharmaceutical molecules are chiral, and their pharmacological activity and side effects can vary significantly between enantiomers. Therefore, precise control over the purity and enantiomeric ratio of chiral drugs is crucial in drug development and usage.

As shown in Fig. 8H, Yin and Xie [132] synthesized carboxylated SWCNTs in a strong acid environment and functionalized them with d- and l-cysteine using the EDC-NHS method. Based on the chemically modified SWCNT films, they developed a metamaterial sensor composed of asymmetric double antenna resonators (ADARs). This sensor exhibited 2 resonances with distinct quality factors in the THz range. SWCNTs served dual functions in this system: They enhanced sensitivity in the THz region and acted as a chiral stationary phase that selectively interacted with one enantiomer of the chiral molecules. By analyzing the optical responses of the chemically modified SWCNT-based THz metamaterial, the sensor could successfully identify chiral molecules. Experimental results demonstrated that the resonance with a lower *Q*-factor was more suitable for sensing in aqueous solutions, exhibiting enveloped resonance characteristics. The SWCNT-based metamaterial sensor displayed high selectivity for detecting chiral compounds in aqueous environments, providing a simple platform for the rapid, cost-effective, and straightforward production of chiral molecule sensors. Using the SWCNT-based ADAR microfluidic system, precise chiral detection of d- and l-tartaric acid was achieved.

Molecular dynamics simulations further revealed the detection mechanism by illustrating differences in hydrogen bonding and contact numbers between chiral molecules and the modified SWCNTs. The results showed that tartaric acid molecules with an opposite configuration to cysteine were more likely to form hydrogen bonds, while chiral molecules with the same optical rotation exhibited weaker intermolecular forces. This SWCNT-based ADAR microfluidic system offers an efficient, noncontact sensing platform for quantitative concentration detection and enantiomeric recognition of chiral biomolecules. Future works should focus on achieving absolute chiral molecule identification and extending the system to a broader range of molecular properties.

### All-dielectric THz metasurface biosensors

In recent years, all-dielectric THz metasurfaces, as an emerging material system, have gradually become a research hotspot in the field of THz wave modulation, imaging, and sensing due to their low loss, high *Q*-factor, and excellent stability [133–136]. In this section, we will focus on reviewing the latest research progress of typical all-dielectric metasurfaces in THz sensing in recent years. By analyzing a series of new THz metasurface structures based on different all-dielectric materials (such as silicon, zirconium dioxide, and lithium tantalate), including gratings, silicon pillar arrays, triangular prism tetramers, and microsphere arrays, the interaction between THz waves and target molecules is effectively enhanced [137]. We will explore its performance in detecting target substances such as biomolecules, tumor markers, and amino acids. Some studies also introduce functionalized AuNPs to further enhance the recognition ability of biological analytes. These studies not only demonstrate the great potential of all-dielectric metasurfaces in improving detection sensitivity and selectivity but also provide new ideas and methods for the design and application of future THz sensors.

**Fig. 9.**
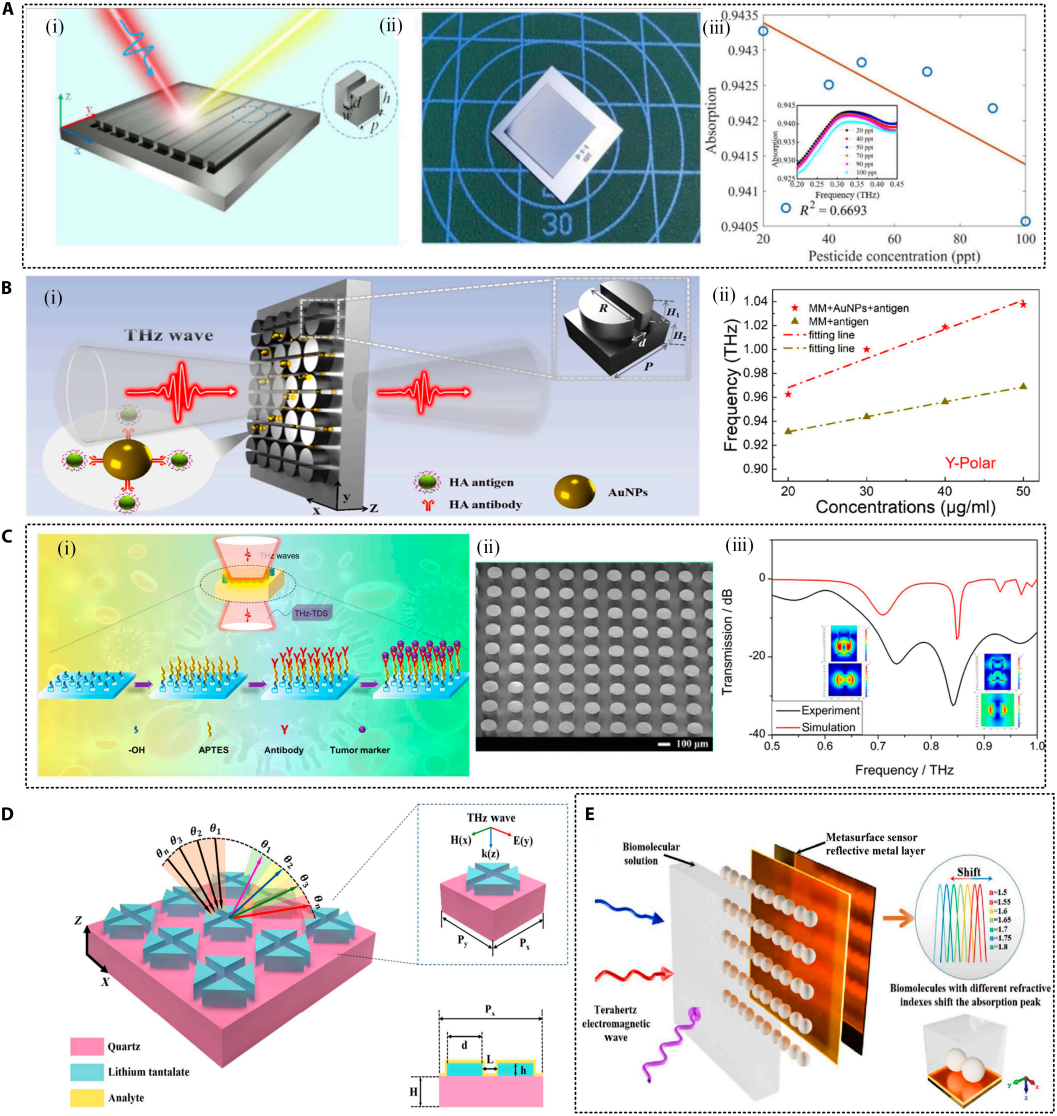
THz metasurface sensor based on dielectric materials. (A) THz metasurface based on N-type silicon. (i) Schematic diagram of multi-band metasurface absorber, (ii) optical photo, and (iii) relationship between spectral intensity and chlorpyrifos concentration [138]. (B) (i) Schematic diagram of the all-dielectric metasurface composed of half-cylinder array. (ii) Relationship between the frequency shift of *f*_2_ and the sample concentration when *y*-polarization is applied [139]. (C) Silicon pillar array THz metamaterial biosensor that can be used for tumor marker detection. (i) Schematic diagram of device surface specific modification, (ii) SEM image of the silicon pillar array, and (iii) simulated and experimental transmission spectra of the all-dielectric metasurface sensor [140]. (D) All-dielectric metasurface based on lithium tantalate triangular prismatic tetramer [141]. (E) All-dielectric metasurface based on zirconium dioxide microspheres [20].

In 2021, Yue et al. proposed an all-dielectric THz multi-band absorption metasurface based on an N-type silicon grating structure, as shown in Fig. 9A [138]. Its application potential in the detection of trace pesticide chlorpyrifos was systematically studied. The device is constructed using a periodic silicon grating, and 3-band and 4-band resonant absorption are achieved by adjusting the etching depth, covering 0.2 to 2.5 THz. The absorption rate at multiple resonance points exceeds 91%, and the highest absorption can reach 99.99%. The *Q* value is as high as 12.64, showing excellent spectral selectivity and resonance enhancement capabilities. By combining finite element simulation with coupled-mode theory (CMT), it is revealed that its resonant absorption originates from the synergistic excitation of SPR and Fabry–Perot-like (GPR) modes. In addition, the device has good dynamic adjustability. By adjusting the carrier concentration of silicon through pump light excitation, an absorption modulation depth of up to 51.91% is achieved, showing its application potential in the direction of tunable THz functional devices. The simulation results show that the resonant frequency and absorption amplitude of the device can be adjusted according to the refractive index of the environment, with a maximum sensitivity of 414 GHz/RIU and a maximum figure of merit (FOM) value of 15.86/RIU. The experimental results show that the device can be used for trace detection of the pesticide chlorpyrifos and can achieve efficient detection in the range of 20 to 100 parts per trillion (ppt). This work provides a new idea for realizing highly sensitive, highly selective, and tunable all-dielectric THz biochemical sensors. On this basis, subsequent research further focused on improving the selectivity and adaptability of biorecognition, and proposed a THz immunosensing platform that synergistically constructs an all-dielectric metasurface with functionalized AuNPs. In 2022, Shi et al. [139] proposed a THz immunosensor combined with functionalized AuNPs for specific recognition and highly sensitive detection of human influenza hemagglutinin tag protein (HA antigen), with a detection concentration as low as nanomolar. As shown in Fig. 9B, the designed cracked half-cylinder array structure has an excellent local enhancement effect of the electromagnetic field and exhibits double resonance characteristics in the range of 0.4 to 1.0 THz. Experiments show that after the introduction of AuNPs, the sensing sensitivity of this method is increased to 2.96 GHz·ml/nmol, which is 2.66 times higher than that of the unmodified system, and the detection limit is 1.05 nmol/ml. At the same time, it can effectively distinguish HA antigen from other nonspecific proteins, verifying its excellent immune-specific recognition ability. In addition, through comparative experiments with 3 other nonspecific proteins, the specific recognition ability of the sensor for HA antigen was demonstrated. This sensor design that combines nanomaterials and all-dielectric metasurfaces not only improves detection sensitivity but also achieves specific detection, providing new ideas and methods for the development of all-dielectric THz metasurface biosensing technology. In 2024, Men et al. [140] further expanded the application of all-dielectric metasurfaces in the field of biomedical detection and proposed a THz metasurface biosensor based on silicon pillar arrays for ultrasensitive detection of tumor markers CA125 and HER2, as shown in Fig. 9C. Unlike existing studies, in this work, specific detection of tumor markers was achieved by antigen–antibody specific modification on the surface of the silicon pillar array. Through surface modification technology, antibodies for CA125 and HER2 were immobilized on the silicon pillar array, enabling the sensor to specifically recognize and bind to the target tumor markers, thereby significantly improving the specificity and sensitivity of the detection. The experimental results show that the sensor has a detection limit of as low as 1 ng/ml for CA125 and a detection limit of as low as 0.1 ng/ml for HER2, and exhibits good linear response in the concentration range of 1 ng/ml to 10 μg/ml. This sensor design based on all-medium metasurfaces and antigen–antibody specific modification not only improves the detection sensitivity but also realizes specific detection, providing new ideas and methods for the development of THz biosensing technology, especially in the field of early cancer diagnosis, which has broad application prospects.

About other all-dielectric material systems, in 2024, Xu et al. [141] proposed a new all-dielectric metasurface THz molecular fingerprint sensor for detecting trace amounts of cinnamoylglycine, a urine biomarker associated with gestational diabetes. As shown in Fig. 9D, the core of the sensor is a periodic array of tetragonal lithium tantalate triangular prisms placed on a quartz substrate. The physical mechanism was analyzed by the finite-difference time-domain (FDTD) method, and the results showed that the metasurface has a *Q*-factor of up to 231 and a FOM of 609, which can significantly enhance the molecular fingerprint signal. By changing the incident angle, the sensor can capture a wide envelope curve corresponding to the absorption resonance frequency of the analyte, thereby achieving high-sensitivity detection of trace analytes with a detection limit as low as 1.23 μg/cm^2^. In this study, by adjusting the incident angle of the THz wave, sensitive responses to different polarization states were achieved; especially, the *y*-polarized wave at an incident angle of 37° was highly matched with the absorption peak of cinnamoylglycine (0.487 THz). In addition, through the angle scanning strategy, the sensor can obtain rich spectral information, further improving the detection ability of trace analytes. The experimental results show that the sensor has a good linear response to cinnamoylglycine layers of different thicknesses, and the detection limit is as low as 1.23 μg/cm^2^, showing excellent sensitivity and specificity. Furthermore, in 2024, Gao et al. [20] designed a single-ball structure metasurface sensor based on zirconium dioxide microspheres, and further improved the stability and reliability of the device by optimizing the structure to a double ball. As shown in Fig. 9E, the working principle of this metasurface sensor is based on Mie resonance, which identifies amino acid solutions with different refractive indices by changing the absorption peak position of the incident THz wave. The performance of the sensor was simulated and optimized using computer simulation technology (CST), focusing on the performance of the sensor in high mode. The experimental results show that the single-ball structure sensor exhibits the best performance in the third mode (the third level of Mie resonance), with a *Q*-factor of 657.93, a sensitivity of 114.75 GHz/RIU, and a FOM of 28.25. However, too high a *Q* value may lead to a decrease in sensor stability. To solve this problem, a double-ball structure sensor was designed in this work. By optimizing the microsphere spacing and other parameters, the *Q* value of the double-sphere structure sensor was reduced to 451.87, the sensitivity was increased to 135 GHz/RIU, and the FOM reached 42.19.

In summary, the all-dielectric THz metasurface sensor has shown broad application prospects in the field of biosensing due to its advantages of low loss, high sensitivity, high *Q* value, simple structure, and easy integration, especially in the detection of biomolecules, biomarkers, and biomedical diagnosis.

## Metasurface Applications in the Microwave Wireless Communication

### Metasurfaces in low-cost wireless communication devices

In 5G communication scenarios, users can experience high transmission rates and large channel capacity. However, due to the presence of obstacles such as buildings or trees, multipath effects are often unavoidable, which significantly reduces the robustness of the communication link. Additionally, IoT systems consisting of satellites, aerial vehicles, sensors, and other components have also gained widespread attention due to their intelligent management and automated control capabilities [142,143]. The stability of the abovementioned application scenarios relies heavily on multi-beam technology. Multi-beam antennas can reduce multipath effects while maintaining high transmission rates, and they are capable of serving multiple users, offering good coverage. One of the representatives of antennas that can dynamically switch between multiple beams is the phased array.

**Fig. 10.**
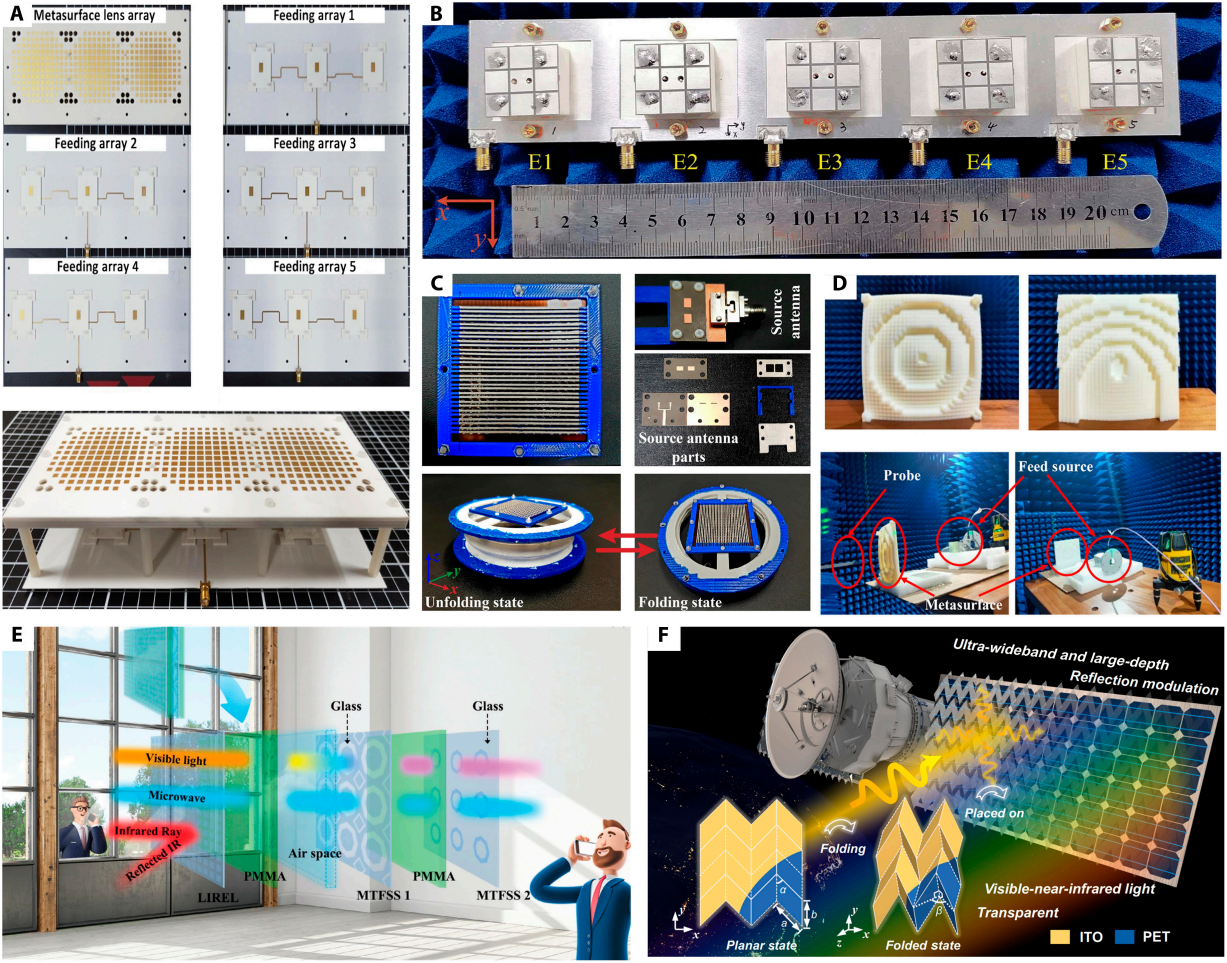
Application of metasurfaces in low-cost antennas. (A) Low-cost MLA fed by a phased array [146]. (B) Hybrid metasurface reconfigurable wide-angle beam-scanning phased array [148]. (C) Foldable low-cost metasurface lens antenna [152]. (D) 3D-printed metasurface for generating a Bessel beam [156]. (E) Schematic of a metasurface glass that can be used for wireless communication and energy saving [165]. (F) Origami metamaterial configuration scheme for solar panels that can be used for satellites [166].

Phased arrays offer advantages such as high flexibility, high gain, and wide beam coverage, but they require the use of numerous phase shifters, which leads to issues such as high-power consumption, high cost, and complex hardware circuits [144,145]. To reduce cost and enable broader applications of multi-beam technology, studies have implemented low-cost metasurfaces as electromagnetic lenses to assist phased arrays in achieving beamforming and beam control functions. Figure 10A shows a metasurface lens array (MLA) fed by a simple phased array. Compared to a typical single large-aperture metasurface lens antenna, the proposed MLA reduces the focal length to one-third and uses only 3 phase shifters across a 7.2*λ*_0_ aperture. This design reduces the thickness of the lens antenna and the number of phase shifters, significantly lowering the cost and allowing for broad potential applications in various communication systems [146]. In addition to being used with phased arrays, metasurfaces can also be combined with reconfigurable antennas to form hybrid metasurfaces, thereby enhancing antenna bandwidth or improving beam steering capabilities. This approach also presents the advantage of low cost and excels in reducing the number of elements and simplifying circuit complexity. In [147], Sheng et al. proposed a multi-beam antenna based on a low-cost reconfigurable hybrid metasurface. The antenna consists of a feeding source and a transmission hybrid metasurface, where the metasurface contains units with 3 different amplitude and phase responses. By using p-i-n diodes loaded on the metasurface, the antenna can switch between 2 and 4 radiated beams. This design offers both compact structure and excellent radiation performance. Wang et al. [148] proposed a miniaturized, broadband hybrid metasurface antenna based on the Huygens’ principle, as presented in Fig. 10B. By utilizing reconfigurable units, the authors successfully achieved wide-angle beam scanning. The antenna array’s cost was reduced through the use of a simple structure and fewer units. During the experiment, a 5-element phased array with a continuous beam scanning range of ±70° was fabricated and measured, validating the advantages of the proposed compact structure and wide beam scanning capability.

In addition to solutions such as reducing the number of array elements and simplifying the structure, additive manufacturing technology, also known as 3D printing, can further reduce production costs. Due to its environmental friendliness, low cost, and ability to create complex structures, additive manufacturing is widely used in the production of microwave devices. Similarly, using 3D printing technology to manufacture dielectric metasurfaces not only reduces the cost but also minimizes conductor losses caused by metallic patterns, making it highly beneficial for large-scale, low-cost metasurface applications [149–151]. Shrestha et al. [152] proposed an antenna that integrates a 3D-printed phase-shifting metasurface with a horn antenna. This antenna shows improvements in directivity, realized gain, and aperture efficiency in the 10- to 18-GHz operating frequency band, and it weighs only 345.37 g, making it suitable for broadband and high-gain wireless communication systems. Figure 10C shows a 3D-printed broadband millimeter-wave metasurface lens antenna, where the meta-atom has orthogonal polarization conversion properties and can adjust both amplitude and phase. The peak gain of the antenna at the operating frequency is 18.3 dBi, with a 10-dB impedance bandwidth of 38%, demonstrating good performance. Furthermore, the lens and the source antenna are connected through a 3D-printed foldable structure, which allows the size to be further reduced during standby mode. This antenna offers advantages of low cost, miniaturization, and rapid manufacturing [153].

Additive manufacturing has also been widely exploited to implement all-dielectric devices designed by transformation optics, a mathematical tool that enables controlling electromagnetic fields with engineered materials presenting spatially varying parameters. As such, a beam steering lens and a focusing lens have been fabricated by 3D polyjet printing and experimentally validated over a broad frequency range in the microwave regime [154]. The same mathematical concept has enabled the fabrication of a flat Luneburg lens antenna with zero focal length through an integrated dielectric and conductive 3D printing manufacturing process [155].

In addition, 3D printing can also be used to manufacture certain metasurfaces that are difficult to process using conventional methods. Qu et al. [156] designed a platform composed of a simple cube as the unit cell and generated a 360° phase shift by adjusting the height of the units, enabling to generate a Bessel beam, as presented in Fig. 10D. The measured results were consistent with the simulation results. This method can also be used to achieve functions such as band-stop frequency selection, polarization conversion, and beam deflection with metasurfaces [157–159]. A fully metal metasurface antenna composed of subwavelength units, where a surface wave is gradually transformed into a leaky wave, has been successfully achieved through additive manufacturing for an operation at 32 GHz [160]. Some technologies even enable 3D printing of alloys, further enhancing the feasibility of manufacturing complex structures [161–164].

Existing programmable metasurfaces (RIS/IRS) are predominantly utilized in the sub-6G frequency range. However, challenges such as low phase modulation precision (1-bit), limited array scale, and integration difficulties at higher frequencies constrain their ability to enhance millimeter-wave (mmWave) communication performance. The mmWave spectrum (e.g., 24 to 30 GHz) is crucial for high-bandwidth 6G communication, yet achieving large-scale array design, multi-user dynamic beamforming, and efficient hardware integration remains a significant challenge. It is worth noting that microwave metasurfaces for wireless communication still face several challenges in practical applications, such as the trade-off between energy efficiency and wireless communication performance in traditional low-emissivity (Low-e) glass. Zheng et al. [165] designed a 3-layer structured metasurface glass, consisting of a low-infrared-emissivity layer (LIREL), an air gap layer, and a microwave transmission frequency-selective surface (MTFSS), as shown in Fig. 10E. The core component of this structure is indium tin oxide (ITO) conductive glass, which features high conductivity, high visible light transmittance, and low infrared emissivity. Experimental and simulation results verified the microwave transmission performance of the metasurface glass in the 5G (sub-6 GHz) band. Under vertical incidence, the transmittance exceeded 54.2% in the 2.5- to 5-GHz range and over 70% in the 2.75- to 4.8-GHz range. Even at larger incidence angles, the transmittance remained stable in the transverse magnetic (TM) mode, while it gradually decreased in the transverse electric (TE) mode. Additionally, the emissivity of unetched ITO conductive glass, etched ITO conductive glass, and ordinary glass was measured using a TSS-5X emissometer. The results showed that the etched ITO conductive glass exhibited an emissivity of 0.32, demonstrating low infrared radiation characteristics. Further experiments revealed that traditional Low-e glass significantly shields communication signals, whereas the metasurface glass effectively transmits 5G (sub-6 GHz) signals, ensuring normal wireless communication functionality. Moreover, a comparison of the thermal insulation performance of ordinary glass, unetched ITO conductive glass, and etched ITO conductive glass indicated that the latter exhibited superior thermal insulation, with a significant temperature difference compared to ordinary glass, further confirming its excellent heat insulation properties. This metasurface glass demonstrates excellent microwave transmission performance in the 5G band while maintaining low infrared emissivity, effective thermal insulation, and high visible light transmittance. It successfully addresses the trade-off between energy efficiency and wireless communication in traditional Low-e glass, providing an innovative solution for integrating energy-saving architecture with wireless communication technology. It holds promising applications in commercial building glass, residential glass, and high-speed railway train windows.

Furthermore, existing dynamic devices suffer from narrow bandwidth, limited modulation range, and high costs. To overcome these limitations, Song et al. [166] proposed a metamaterial based on origami technology to achieve ultrawideband and deep-depth reflection modulation. As illustrated in Fig. 10F, the metamaterial consists of a 0.125-mm-thick PET substrate and a 20-nm-thick ITO thin film, offering excellent mechanical properties and optical transparency. Experimental results indicate that the metamaterial exhibits strong reflection near 0 dB in its planar state, while in the folded state, it achieves weak reflection below −10 dB across the 4.96- to 38.8-GHz frequency range, with an average modulation depth of 11.53 dB. Additionally, the metamaterial maintains high transmittance in the visible-to-near-infrared spectrum, with an average optical transmittance exceeding 87.2%. Under oblique incidence at 60°, the metamaterial retains its ultrawideband reflection modulation performance. Furthermore, the folding and unfolding process demonstrates its excellent mechanical properties and foldability. Experimental validations confirm the metamaterial’s effective reflection modulation capabilities across microwave, visible, and near-infrared frequencies, as well as its stability under varying incident angles and polarization conditions. With advantages such as high transparency, lightweight structure, and low cost, this origami-based metamaterial provides a novel solution for satellite communications and optical window-based mobile communication management.

### Reconfigurable metasurfaces for wireless communication

In modern society, with the large-scale commercialization of 5G technology and the emergence of various new application scenarios, the demand for wireless communications continues to grow. Under the constraints of limited spectrum resources and channel capacity, improvement of communication quality has become an urgent issue that needs to be addressed. In fact, in a deployed communication system, there exists unused electromagnetic energy, which is the energy dispersed in the surrounding space. If this energy can be utilized, it would effectively enhance the signal-to-noise ratio (SNR) and create a higher-quality communication environment. In the process of innovating and optimizing metasurfaces, it has been discovered that incorporating tunable electronic components to metasurfaces can equip them with reconfigurable properties. By using PIN diodes, varactor diodes, and other components with tunability features, metasurfaces gain exceptional capabilities for dynamically controlling electromagnetic waves, thus being referred to as reconfigurable intelligent surfaces (RISs). It is widely recognized that RIS, due to its low profile, ease of deployment, and low cost, has the ability to reshape the electromagnetic environment [167,168]. By placing RIS at suitable locations near communication systems, energy can be redirected and steered toward the receiver, enhancing the SNR and effectively increasing the usable energy in the environmental free space, eliminating the need to match it with any module of the communication system, greatly enhancing the flexibility of RIS.

**Fig. 11.**
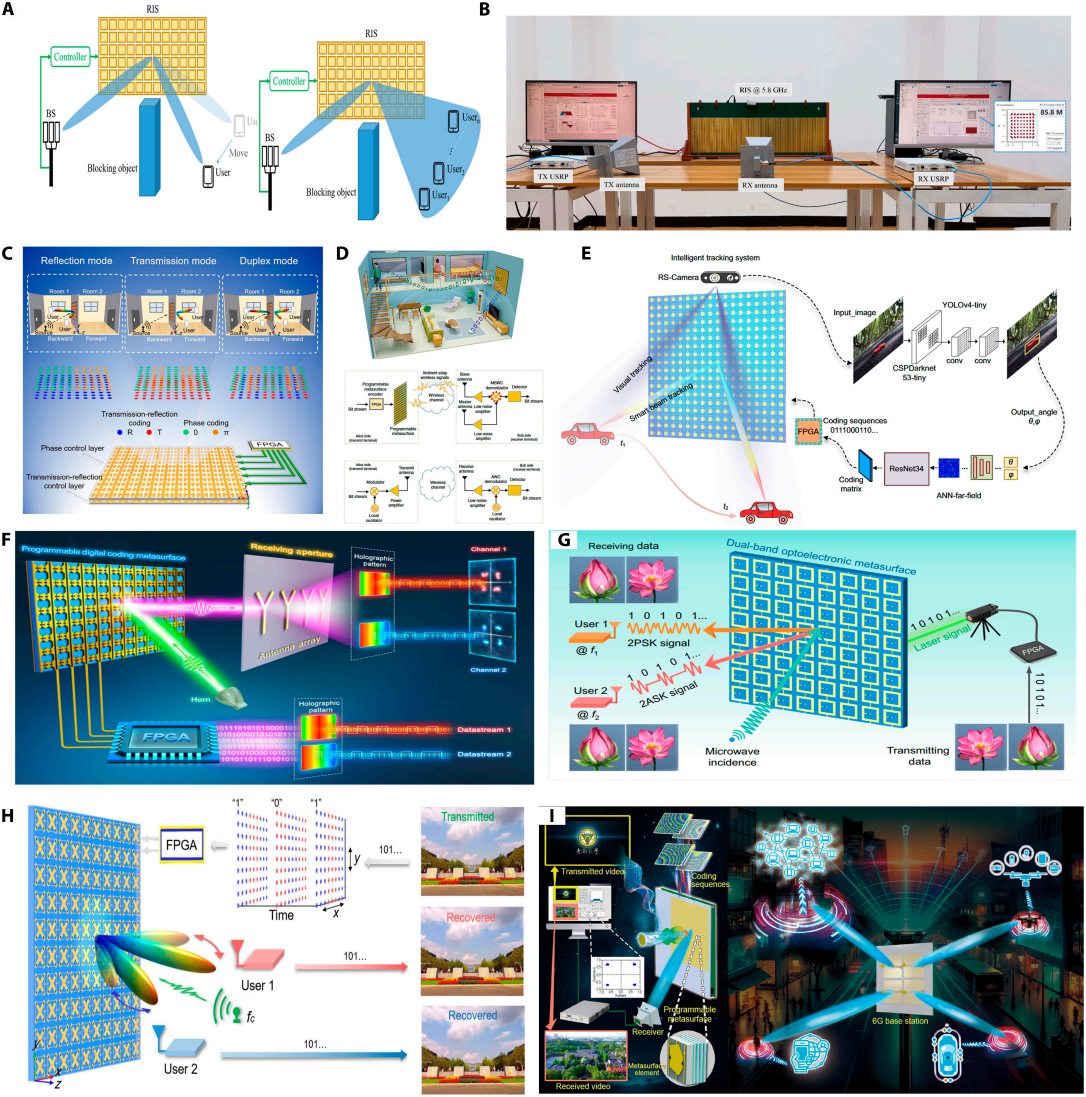
RIS for wireless communication. (A) Two scenarios of RIS-assisted wireless communications [169]. (B) Prototype of the RIS-aided wireless communication system [170]. (C) IOS [173]. (D) Scenario and block diagram for the proposed MBWC in a typical indoor environment [67]. (E) Intelligent target tracking system based on RIS [178]. (F) Schematic diagram of holographic MIMO scheme based on PDCM and electromagnetic information theory (EIT) [179]. (G) Schematic of a laser to microwave wireless transmission system based on dual-frequency time-domain photoelectric metasurface [180]. (H) Schematic diagram of a broadband wireless communication system based on a time-varying polarization conversion metasurface [181]. (I) Schematic diagram of a novel intelligent beamforming programmable metasurface system [182].

Tang et al. [169] have demonstrated the role of RIS in improving the transmission efficiency of wireless systems and developed free-space path loss models for RIS-assisted wireless communication in different scenarios, laying a theoretical foundation for practical applications, as shown in Fig. 11A. Di Renzo et al. [170] have also analyzed the new communication-theoretic models required for RIS design and the challenges that need to be addressed for its large-scale deployment. In terms of RIS prototype design, Fig. 11B shows a RIS with 1,100 controllable elements operating at 5.8 GHz and developed a highly efficient algorithm to establish a RIS-assisted wireless communication link. During indoor measuring, the RIS provided a 26-dB power gain compared to the baseline case, demonstrating the potential of RIS-assisted communication systems [171]. The angular sensitivity of RIS affects communication systems that rely on the reciprocity of wireless channels. To address this issue, Liang et al. proposed an angle-insensitive 3-bit RIS. Both simulations and measurements demonstrated that this RIS provides stable phase shifts over a wide incidence angle range of 0° to 60° and possesses angular reciprocity [172].

Most current RISs only support either reflection or transmission modes, which limits signal enhancement to half of the environment during wireless communication. To meet the needs of more users and extend the range of efficient communication, Hu et al. [173] proposed an intelligent programmable omni-surface (IOS), and achieved full-dimensional communication. The work also discusses application scenarios of IOS-assisted wireless communication, including coverage extension, interference cancellation, secure communications, as well as sensing and localization, as shown in Fig. 11C. In [174], the flexibility of the IOS has been further enhanced, supporting reflection mode, transmission mode, and duplex mode with the same polarization and frequency. It is capable of independently achieving beam scanning in both modes, providing ubiquitous full-space service coverage for multi-user wireless communication applications. The IOS demonstrates excellent performance in a variety of specialized application scenarios.

The aforementioned work has effectively demonstrated that RIS can significantly enhance the SNR and expand the efficient communication range of wireless communication systems under limited spectrum resources and channel capacity. Moreover, RIS may also contribute to lightweight signal transmitters or secure communications. Transmitters often require heavy and expensive components such as nonlinear mixers and power amplifiers, which reduce the flexibility of base stations and make them less suitable for large-scale Internet of Things (IoT) applications and the deployment of intelligent wireless communication environments. Moreover, as communication environments become increasingly complex, secure communication has gained growing attention. To address these pressing issues, research groups have started exploring the use of RIS to modulate information in various forms. In [175], Dai et al. proposed a prototype system for quadrature phase-shift keying (QPSK) wireless communication based on time-domain digital coding metasurfaces. By dynamically altering the reflection characteristics of the metasurface in real time, QPSK modulation of the baseband signal is achieved when the carrier wave reflects off the metasurface. High data-rate video transmission was demonstrated experimentally. Zhang et al. [176] proposed a wireless communication scheme utilizing a RIS to achieve space-division and frequency-division multiplexing. By using a specially designed space–time coding matrix, different users at various locations can simultaneously receive information without the need for mixing or digital-to-analog conversion. In the experiments, when the users were in asymmetric directions, different images were successfully transmitted to each user, demonstrating the advantage of space-division multiplexing, i.e., directional signal transmission. Conversely, when both users were positioned in the same location, 2 distinct images were still separately delivered to each user, showcasing the advantage of frequency-division multiplexing, i.e., mutual isolation of different channels. In addition to modulating actively generated carrier waves to transmit information, RIS can also modulate scattered waves in the environment while achieving high data rates and strong information security. Figure 11D shows a massive backscatter communication (MBWC) concept, utilizing RIS to modulate ambient 2.4-GHz Wi-Fi signals. Through a specially designed method, the information encoded by the RIS is transmitted to users with high SNR and robust security. In the experimental validation, a 3-channel QPSK modulation scheme was designed, where 3 monochromatic patterns were successfully transmitted to 3 different users with excellent communication quality. This communication scheme provides a novel perspective on the role of RIS in wireless communications [67]. Moreover, various active modulation schemes based on RIS have been proposed, providing new perspectives for the future development of RIS.

Most current RIS platforms rely on manual control, such as enhancing signals within a designated area. However, when users move out of the specified area or have other demands, the advantages of RIS-assisted wireless communication systems diminish. Therefore, the self-adaptive capability of RIS is crucial. In [177], She et al. designed a RIS capable of self-adaptive electromagnetic manipulation based on different incident electromagnetic wave information. This metasurface consists of a sensing module, a field-programmable gate array (FPGA) control platform, and executing materials. The FPGA processes the power intensity of the incident wave received by the sensors, enabling the metasurface to switch between transmission, reflection, and tunable absorption functions, thus forming a complete self-adaptive system. In addition, Li et al. [178] proposed another solution for target tracking in wireless communication, as shown in Fig. 11E. This system leverages computer vision and convolutional neural network (CNN), along with a dual-polarized metasurface, to achieve automatic monitoring of moving targets. Real-time wireless communication is implemented using a metasurface integrated with a pretrained artificial neural network (ANN). In the 3 demonstrated experiments, the system successfully achieved the identification and detection of moving targets, detection of radio frequency (RF) signals, and real-time wireless communication. This solution opens a new pathway for adaptive wireless communication systems.

As is well known, the novel wireless communication scheme of holographic multiple-input multiple-output (MIMO), which integrates spatial continuous control of the electromagnetic field and multi-antenna technology, can achieve higher spatial degrees of freedom and channel capacity within limited physical space, thereby overcoming the limitations of traditional MIMO systems. As shown in Fig. 11F, Shao et al. [179] proposed a holographic MIMO scheme based on programmable digital coding metasurface (PDCM) and electromagnetic information theory. Theoretically, orthogonal current-field modes of the free-space radiation operator were extracted through the Hilbert–Schmidt decomposition, and the system’s effective degree of freedom (EDoF) was quantified based on geometric parameters, yielding a value of 2.16. This further validated the accuracy of the theoretical model and the multi-channel communication capability of the proposed holographic MIMO system. In the design, a 2-bit phase-modulated PDCM (24 × 16 units, operating at 4.95 GHz) was employed, with real-time control of the encoding mode via FPGA to map QPSK symbols to orthogonal spatial channels. The received signal was demodulated through inner product operations. Experimental validation showed that the dual-channel QPSK constellation symbols were distinguishable, and performance remained stable in non-line-of-sight scenarios (with negligible constellation diagram offset). The measured EDoF matched the theoretical value, confirming the spatial orthogonality. Compared to traditional MIMO, the PDCM approach eliminates the need for dense RF components, reducing costs by 50%. Moreover, by subwavelength control (unit size 0.25λ × 0.25λ), it approximates a continuous aperture and suppresses mutual coupling effects. In comparison with existing PDCM studies, this paper is the first to utilize spatial diversity to realize a single-user dual-channel system, with a receiver aperture spacing of only 2.475λ (compared to 15λ in traditional schemes), which is more aligned with practical scenario requirements. To further reduce the complexity, power consumption, and cost of traditional electronic circuit systems, Zhang et al. [180]. proposed a novel approach. They developed a dual-frequency time-domain optoelectronic metasurface-based laser-to-microwave wireless transmission system and experimentally verified its potential for high-speed data transmission, as shown in Fig. 11G. By integrating an optoelectronic metasurface with a photodetection circuit, the system enables phase and amplitude modulation of microwave signals controlled by laser signals, achieving efficient laser-to-microwave signal conversion. Experimental results show that the system can modulate microwave signals at 3.5 and 5.0 GHz in terms of phase and amplitude, respectively, and successfully achieve a data transmission rate of 2.5 Mbps, demonstrating its effectiveness in wireless communication. Compared to conventional communication systems, this novel approach not only offers higher flexibility and lower complexity but also provides a hybrid optical-electronic communication scheme by simultaneously modulating laser and microwave signals, improving transmission efficiency and data rates. Although signal quality degradation was observed at higher data rates, the system overall outperforms traditional solutions, showcasing the application potential of dual-frequency optoelectronic metasurfaces in future wireless communication systems. Future advancements can be achieved by introducing higher-order modulation schemes or optimizing the metasurface design to further enhance data rates and system stability.

Hu et al. [181] proposed a broadband wireless communication system based on a time-varying polarization conversion metasurface, as shown in Fig. 11H. By leveraging spatiotemporal joint modulation, this system enables efficient information encoding and transmission. Unlike traditional wireless communication systems, this approach eliminates complex RF links and directly utilizes a programmable metasurface to control the reflection phase and polarization direction of electromagnetic waves, thereby encoding digital information. Experiments demonstrate that the system operates stably within the 3.7- to 5.1-GHz frequency band and can simultaneously transmit directional beams to multiple users, achieving regional signal enhancement and signal leakage suppression. By employing variable spatial encoding patterns, the system dynamically adjusts communication beams based on user locations, improving transmission efficiency and enhancing information security. Additionally, it supports high-speed data transmission based on phase modulation offering stronger anti-interference capabilities compared to traditional amplitude modulation schemes. Further experimental validation confirms the system’s multi-user communication capability, as transmitted color image information is successfully reconstructed at different user terminals. With its broadband characteristics, low-power architecture, and reconfigurability, this system provides a novel solution for next-generation wireless communication. In the future, this approach can be integrated with millimeter-wave or THz technologies to enable intelligent, adaptive wireless communication at higher frequency bands, driving the evolution of wireless networks from static to intelligent and dynamic systems. In addition, Zhang et al. [182] proposed an intelligent mmWave base station for 6G applications based on programmable metasurfaces. As illustrated in Fig. 11I, the system consists of a feed source, a programmable metasurface, and a control board, enabling precise and wide-ranging 2D beamforming. The programmable metasurface comprises a 30 × 30 array of metasurface elements, each embedded with 2 PIN diodes, achieving a 2-bit phase modulation capability. The control board integrates an FPGA core and 38 power driver chips, featuring 1,800 independent output ports to control all PIN diodes. By carefully designing the phase distribution on the metasurface, the array can be dynamically reconfigured to realize the desired electromagnetic functionalities. Experimental results demonstrate that the reflected beam can be scanned from *θ* = −70° to 70° in both the *φ* = 0° and *φ* = 45° planes, achieving a gain of approximately 23 dBi with a sidelobe level of around −12 dB. The measured results exhibit strong agreement with theoretical calculations and simulations, validating the system’s high performance. Furthermore, wireless communication experiments conducted in a real indoor 6G smart base station scenario confirm that the beamforming system effectively enhances or suppresses signals in specific directions, demonstrating its potential as an auxiliary device for base stations.

### Multifunctional integrated metasurfaces for wireless communications

With internet applications expanding explosively, proliferation of IoT devices, and widespread adoption of 5G technology, the global demand for wireless communication continues to rise. However, spectrum resources, as limited natural resources, cannot meet the increasing communication demands. Moreover, the widely used 5G networks need to support various application scenarios, including low latency, wide bandwidth, and multi-user connectivity [183]. Under such diversified requirements, the network environment has become more complex, intensifying the need for highly integrated devices with multitasking capabilities. Currently, metasurfaces have already achieved multiple functions in the microwave band, such as beam scanning, polarization conversion, orbital angular momentum (OAM) beams, and radar cross-section (RCS) reduction [184–188]. However, metasurfaces that can only perform a single function cannot meet the demands of the aforementioned wireless communication environment. To improve the information processing efficiency of metasurfaces while reducing network deployment costs and complexity, research works about multifunctional metasurfaces have been initiated. It is important to note that the multifunctional metasurfaces discussed here should be capable of independently controlling variables in different dimensions, such as spectrum, polarization, and power. Metasurfaces that only achieve similar functions will not be discussed.

**Fig. 12.**
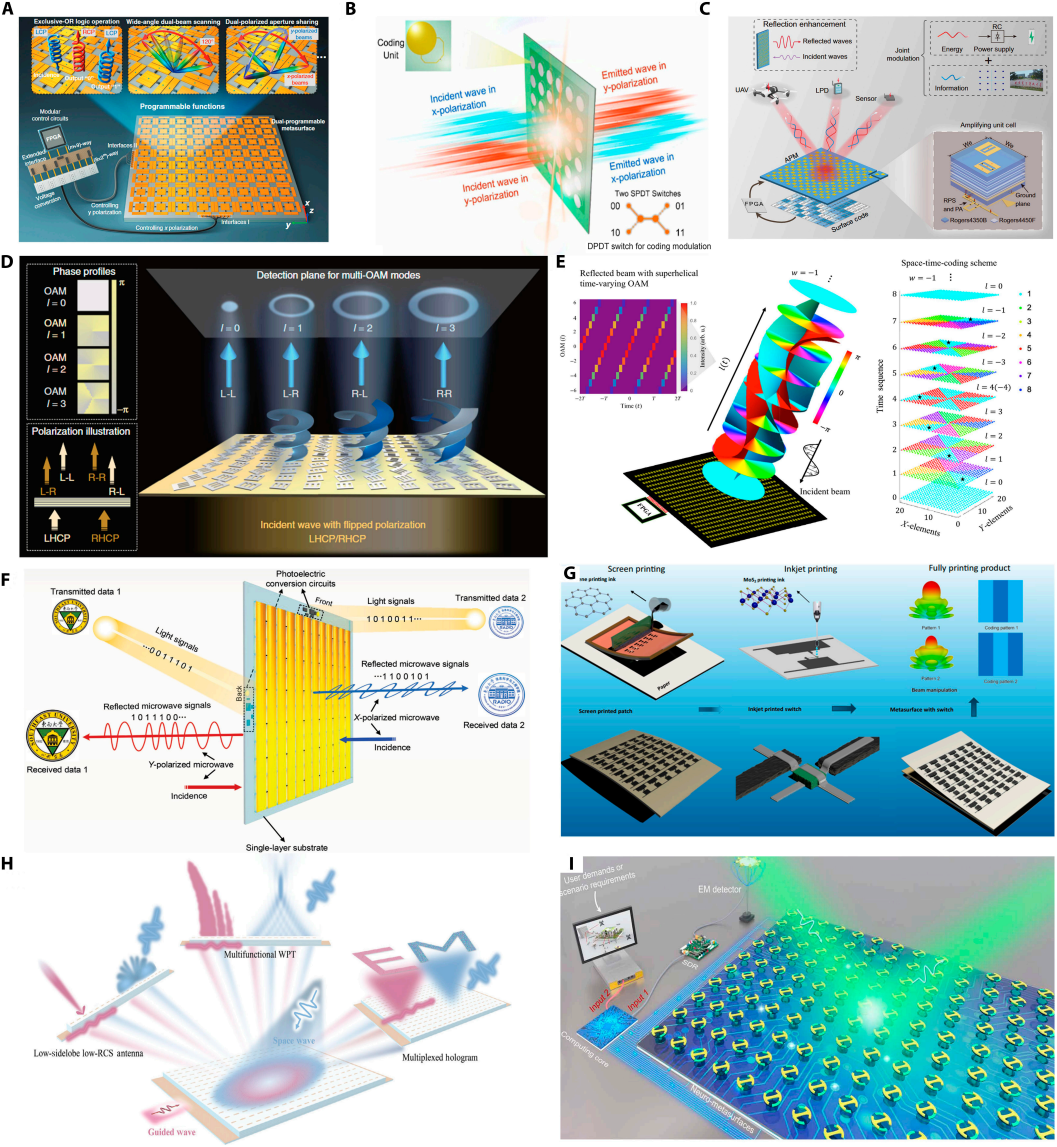
Multifunctional metasurface. (A) Programmable metasurface capable of independently controlling orthogonally polarized electromagnetic waves [189]. (B) Metasurface based on SPDT switches [190]. (C) Jointly modulated APM for a SWIPT system [195]. (D) Proposed metasurface for complete phase modulation in quadruplex polarization channels [198]. (E) Generate time-varying OAM beams using metasurfaces [199]. (F) Schematic diagram of a dual-channel optical–microwave link based on a space-polarization-division multiplexed optoelectronic metasurface [200]. (G) All-printed zero-static power MoS_2_ switch coded reconfigurable graphene metasurface [201]. (H) Schematic of a metasurface capable of multiplexing both guided and space waves [202]. (I) Schematic representation of the homeostatic neuro-metasurface architecture [203].

Dual polarization can support 2 independent channels under the same spectrum resource conditions while also enhancing interference resistance. Zhang et al. [189] realized a metasurface capable of independently controlling *x*- and *y*-polarized waves, as depicted in Fig. 12A. Using this metasurface, the authors implemented an exclusive-OR logic platform for dynamically controlling the spin state of circular polarization (CP) waves, a dual-beam scanning antenna system, and a dual-polarized shared-aperture antenna system. This metasurface offers advantages such as wide-angle scanning, high gain, and high cross-polarization isolation, further improving the integration of the metasurface. Figure 12B shows a metasurface based on single-pole double-throw (SPDT) switches, where the switch states are controlled by an FPGA. The incident *x*- and *y*-polarized waves can be modulated into cross- and co-polarized waves. The proposed metasurface can be used as a near-field information encoder, capable of encoding 9-bit binary information in near-field imaging and switching at high speed [190]. Many other works have also achieved the control of dual-linear or dual-circular polarized waves, contributing to alleviating the tension on spectrum resources and expanding channel capacity [191–193].

When metasurfaces are applied to wireless communication assistance or detection systems, their effectiveness is often limited by transmission distance due to inherent losses. Amplifying metasurface has been designed to further improve signal quality. Wu et al. [194] designed a broadband C-band metasurface with a power amplifier. This metasurface achieves a gain of 7.7 to 12.2 dB in the 5- to 6-GHz range and realizes polarization conversion, which extends the operational range of the metasurface to some extent. Wang et al. [195] proposed a jointly modulated amplifying programmable metasurface (APM) as a transmitter for a simultaneous wireless information and power transfer (SWIPT) system, as depicted in Fig. 12C. The proposed unit enables stable 2-bit phase manipulation. The work also introduces a joint modulation strategy that significantly improves the rectifier conversion efficiency and the average output power of the APM. In the experiments, the SWIPT system simultaneously powered a light-emitting diode array and transmitted video signals, showcasing its potential applications in fields like 6G wireless communication systems, IoT, and beyond. In addition, the authors also proposed an amplifying space–time coding metasurface, which further enhances the integration of the metasurface. By leveraging the nonlinearity of the power amplifier, the power intensity at harmonic frequencies is enhanced, thereby extending the transmission distance of the harmonic waves. In the experiments, dynamical switching of the power amplifier is performed according to the proposed space–time coding matrix and harmonic beams are generated with predefined patterns, which can be applied to radar or wireless relay systems [196].

In complex communication systems, besides indicators such as transmission rate and channel capacity, the anti-jamming capability of transmitted signals is also an important factor to consider. OAM possesses 2 key characteristics: orthogonality between modes and mode diversity. These characteristics not only help improve spectrum utilization but also theoretically isolate interference in the form of plane waves. In terms of multi-frequency points, Huang et al. [197] proposed a broadband 2-bit shared-aperture dual-band transreflective metasurface, which independently generates OAM beams at 14.5 to 20.7 GHz and 30 to 40 GHz, with potential applications in multifunctional wavefront shaping. In terms of multi-polarization, Yuan et al. [198] developed a general phase modulation scheme that can simultaneously activate all CP channels and fully utilize energy, as seen in Fig. 12D. By synthesizing chirality-assisted phase response, propagation phase, and Pancharatnam–Berry (PB) phase, the scheme achieves independent decoupling of the components of the Jones matrix, enabling the generation of OAM beams with 4 independent topological charges. The proposed modulation scheme is highly versatile and contributes to multifunctional metasurfaces and OAM applications in channel expansion. In terms of space–time modulation, Fig. 12E shows a space–time coding metasurface to construct time-varying OAM beams and developed a dual-probe measurement technique to facilitate the construction and observation of time-varying OAM beams. This work introduces a new degree of freedom for OAM, which can enhance communication capacity and provide new avenues for time-division multiplexing and information encryption [199]. Metasurfaces, as 2D artificial structures capable of efficiently manipulating electromagnetic waves, have shown great potential in wireless communication. Evolving from basic reflection and refraction control to multifunctional and intelligent wireless communication applications, metasurface technology has undergone significant advancements, including enhanced functional integration and dynamic tunability.

For example, Sun et al. [200] developed an optoelectronic metasurface based on spatial polarization division multiplexing, enabling a dual-channel optical-to-microwave link, as shown in Fig. 12F. This metasurface comprises a dual-polarized, double-sided structure integrated with a high-speed optoelectronic conversion circuit. Each meta-atom consists of 2 orthogonally oriented metallic pattern layers, each embedded with varactor diodes, allowing independent reflection phase responses under different polarization states of incident microwaves. By adjusting the bias voltage of the varactor diodes, dynamic control over the reflection phase is achieved. Additionally, the optoelectronic conversion circuit converts optical signals into electrical signals, thereby modulating the metasurface’s reflection phase in real time. Experimental results demonstrate that varying the light intensity induces substantial reflection phase shifts across different frequencies, enabling binary frequency shift keying modulation. Specifically, as the light intensity increases from 50 lx to 600 lx, the reflection phase shifts from −180° to 120°, achieving a total phase variation of approximately 300° across all measured frequencies. At 5.0 GHz, error vector magnitude (EVM) measurements at different communication rates reveal that while EVM values increase with transmission speed, they consistently remain below −10 dB, indicating reliable communication performance. Even at a transmission rate of 400 kbps, the system successfully decodes both data channels.

Moreover, this metasurface exhibits excellent microwave transmission characteristics in the 5G (sub-6 GHz) band, achieving a reflection efficiency of approximately 67% at 5.0 GHz. By enabling efficient optical-to-microwave conversion, the proposed metasurface integrates the high bandwidth and security of visible light communication with the long-range transmission capability of RF communication, thereby expanding the potential of wireless communication systems. This hybrid communication approach is particularly advantageous in non-line-of-sight scenarios, offering new possibilities for future wireless networks. Traditional metasurface-based functional devices typically employ rigid substrates and complex fabrication processes, limiting their flexibility and scalability for large-scale production. To enable flexible devices capable of bending and folding to adapt to complex shapes and diverse environments while simplifying fabrication, reducing costs, and improving production efficiency, Xiao et al. [201] proposed a fully printed, zero-static-power MoS₂ switch-encoded reconfigurable graphene metasurface for RF and microwave electromagnetic wave manipulation and control, as shown in Fig. 12G.

This metasurface consists of 36 zero-static-power Ag/MoS₂/Ag switches and 36 graphene patches integrated on a flexible substrate. By switching the states of the Ag/MoS₂/Ag switches, each graphene patch can achieve a phase shift of 180°. Experimental results demonstrated that the metasurface, incorporating nonvolatile Ag/MoS₂/Ag switches (switching ratio >10^5^, zero static power consumption), enables efficient dynamic modulation of RF and microwave electromagnetic waves on a flexible paper-based substrate. Through 1-bit encoding control of the 6 × 6 graphene patch array, the metasurface achieves a 180° phase shift at 3.54 GHz, supporting beam shaping (with main lobe directions ranging from 41° to 52°) and RCS reduction (with insertion loss <0.7 dB). The study reveals a switching mechanism based on silver ion migration-induced local phase transition in MoS₂ (2H → 1T) and employs a fully printed fabrication process (graphene sheet resistance of 1.5 Ω/sq, MoS₂ layer thickness of 2.2 μm) to address the power consumption and flexibility compatibility challenges of conventional reconfigurable devices. This design not only enables efficient electromagnetic wave manipulation but also significantly reduces static power consumption, making it highly suitable for applications in wireless communications, sensing, and holographic imaging. The study overcomes the energy consumption limitations of traditional semiconductor switches, laying the foundation for low-cost, flexible metasurface applications. However, the metasurface’s control remains in a predefined static mode (e.g., beam switching) and lacks advanced dynamic adaptability.

Guan et al. [202] designed a metasurface capable of simultaneously multiplexing guided waves and spatial waves, enabling advanced electromagnetic wave manipulation. As shown in Fig. 12H, this metasurface, featuring specially designed subwavelength structures, can independently and concurrently control the complex amplitude of guided waves and the reflection phase of incident spatial waves, effectively mitigating crosstalk issues. Based on this innovative metasurface design, the research team developed 3 devices with advanced electromagnetic functionalities. The first is a low-sidelobe, low-RCS antenna, which employs a Taylor amplitude distribution to control sidelobe levels while utilizing reflection phase distribution to reduce RCS. Experimental results demonstrated that the antenna achieved ±30° beam deflection and −14-dB sidelobe suppression under different polarization incident waves. Additionally, a parabolic phase distribution led to a monostatic RCS reduction of 15 dB (*y*-polarization) and 6 dB (*y*-polarization). The second device is a multifunctional wireless power transfer system capable of both reflective focusing and radiative transmission of energy, making it highly suitable for long-distance power transfer with enhanced efficiency. The third device is a feed-reused hologram, which leverages both guided and spatial waves to enable dual-channel holographic imaging with imaging efficiencies of 75.6% and 60.8%, respectively. These research findings not only highlight the immense potential of metasurfaces in electromagnetic wave control but also provide novel technological pathways for applications in radar systems, wireless communication, and wireless power transfer. By further integrating tunable elements such as diodes and liquid crystals, future metasurfaces are expected to achieve independent and dynamic control of radiation and reflection characteristics, enabling more flexible electromagnetic wave manipulation and driving the widespread adoption of high-performance, multifunctional metasurface devices.

With the development of smart cities and the IoT, the demand for dynamic management of the physical layer in wireless communication (i.e., wireless channels) within complex propagation environments has become increasingly urgent. Traditional approaches rely on high-power-consuming base station hardware and iterative optimization strategies, which struggle to adapt to dynamic environments. Metasurfaces, as artificial electromagnetic interfaces, offer flexible electromagnetic wave manipulation. However, most existing designs are either static or require manual trial-and-error adjustments, making autonomous real-time response unattainable. To address this limitation, Fan et al. [203] introduced a deep learning-driven global inverse design model, endowing metasurfaces with self-sensing and dynamic adjustment capabilities to achieve real-time beamforming in complex environments, such as mobile target tracking and signal coverage optimization. As shown in Fig. 12I, this study integrates mechanically tunable metasurfaces with AI algorithms to establish a closed-loop “perception–decision–action” system, overcoming the constraints of conventional metasurfaces that rely on predefined configurations. Specifically, by selecting 3 different wireless channel characteristics for blind testing, the inverse-designed metasurface achieved classification accuracies of 94%, 97.5%, and 98.25%, respectively. The measured RCS curves closely matched theoretical predictions, validating the reliability of the design. In simulated real-world scenarios, the system dynamically adjusted wireless channels based on pedestrian positions and density, enabling signal enhancement or suppression as required. Additionally, real-time tracking of public transportation demonstrated the system’s accuracy and agility, highlighting its adaptability to complex environments. By integrating deep learning with metasurface technology, this study represents the first realization of autonomous, real-time wireless channel self-management without human intervention. The proposed global inverse design approach, mechanically reconfigurable metasurface, and perception–decision–action system introduce a novel paradigm for future intelligent communication infrastructure, paving the way for energy-efficient and highly flexible 6G communications and IoT development.

### Communication-sensing integrated metasurface for wireless communication

**Fig. 13.**
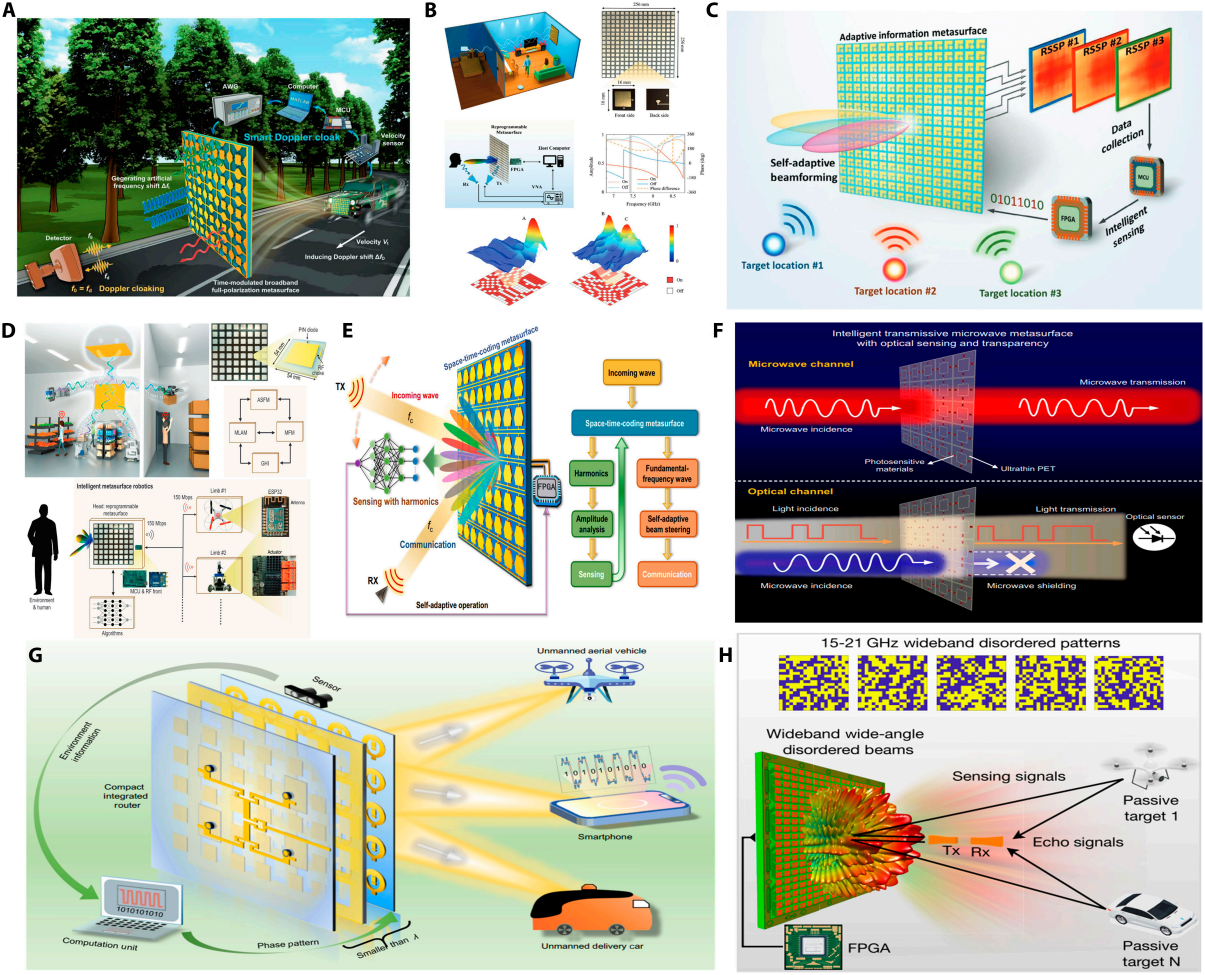
Metasurface with integrated sensors. (A) Smart Doppler invisibility cloak [209]. (B) Microwave speech recognition technology based on programmable metasurfaces [211]. (C) Adaptive metasurface for intelligent target localization and beam tracking [221]. (D) Intelligent indoor metasurface robotics [222]. (E) Schematic diagram of the ISAC scheme based on space–time coded metasurface [232]. (F) Schematic representation of an intelligent transmitted microwave metasurface with optical sensing and transparency [233]. (G) Intelligent wireless power transmission framework based on a 2-bit compact reconfigurable transport metasurface router [234]. (H) Schematic of a multifunctional reconfigurable smart surface for passive object detection [235].

Currently, reconfigurable metasurfaces have demonstrated extraordinary potential in wireless communication, beam control, invisibility cloaks, and imaging [204–208]. In order to achieve higher levels of development, there is a growing interest in making metasurfaces more intelligent so that they can play a crucial role in areas such as the IoT, detection systems, and smart homes. The prerequisite for the intelligence of metasurfaces is the perception and processing of key information from the environment. The ability to actively adjust based on environmental changes without relying on human control is the key to determine whether a metasurface is truly intelligent. Based on this research direction, scholars have started integrating sensors into metasurfaces, with several typical applications shown in Fig. 13. In [209], Zhang et al. integrated motion sensors onto a metasurface to realize a smart Doppler invisibility cloak that operates in broadband and full polarizations, as presented in Fig. 13A. This system can actively change the modulation signal based on the velocity variation of an object to cancel Doppler shifts, which could be applied in intelligent camouflage systems, Doppler radar, and other applications. In [210], Ma et al. proposed a smart metasurface integrated with a gyroscope sensor and a fast feedback algorithm. The metasurface can self-adaptively adjust beam steering or implement multi-beam guidance and other functions by sensing the motion posture. This metasurface is highly expandable and can be integrated with other sensors. Zhang et al. [211] proposed a microwave speech recognition technology based on programmable metasurfaces, as seen in Fig. 13B. The system can focus the electromagnetic wave beam on the speaker’s mouth while also tracking the moving speaker in real time. It utilizes deep learning techniques to interpret the measured biosignals, demonstrating unprecedented accuracy in speech recognition during the process. This work has potential applications in various fields such as intelligent healthcare, smart detection, and more. Meftah et al. [212] has developed an intelligent connected system, based on a programmable metasurface reflector, for dynamic uplink and downlink communication scenarios, where direction of arrival (DOA) of an incoming wave can be estimated and mobile targets can be tracked using global positioning system (GPS) data from a real-time database. Deep learning has later been exploited in such metasurface platform for fast and accurate DOA estimations without the need to rely on traditional systems based on antenna arrays and complex algorithms [213–216].

In addition to collecting environmental information such as spatial movement, velocity, and biosignals, another type of intelligent metasurface is designed to specifically sense and respond to microwave scattering fields. By adapting to the information of the incident waves, these metasurfaces can perform self-adaptive control [217–219]. Since they sense and respond to the same object, such metasurfaces offer higher accuracy and greater specificity. Ma et al. [220] proposed a metasurface with both sensing and response capabilities. The metasurface includes sensing units and execution units, which are used for incident wave sensing and reflecting field manipulation, respectively. The sensing units can detect the power of incident waves in both *x*- and *y*-polarizations. When the power exceeds a threshold, the execution units perform preset functions. This metasurface is the first to combine sensing and response capabilities, advancing the development of intelligent metasurfaces. In [221], Jiang et al. proposed a metasurface capable of simultaneously achieving intelligent target sensing and adaptive beamforming, as presented in Fig. 13C. Each unit of the metasurface serves as a polarization-insensitive electromagnetic power sensor and a 1-bit phase modulator. During the experiments, incident electromagnetic waves were received, sensed, and redirected to a preset direction, with accurate target location estimation. The experimental results were in strong agreement with the simulations. Zhao et al. [222] further extended the concept of intelligent metasurfaces by introducing the idea of intelligent indoor metasurface robotics (I2MR), as depicted in Fig. 13D. I2MR consists of a central robotic “brain” and limbs. The brain is a programmable metasurface with computational capabilities, capable of performing complex sensing tasks and establishing high-quality communication links with the limbs for efficient control. The limbs are composed of mobile vehicles or drones, responsible for executing commands sent by the metasurface. The research demonstrates how the programmable metasurface performs a series of sensing, localization, and communication tasks, with potential applications in various fields.

The metasurface can also be used as a sensor in fields such as healthcare or detection. The work in [223] developed a broadband, low-profile wearable antenna by integrating textiles and metasurfaces, and applied it in a wireless body area network (WBAN). The antenna can test the physiological data of the wearer, facilitating subsequent transmission to mobile terminals or medical monitoring equipment. In addition, other studies have demonstrated the use of metasurface sensors for detecting protein concentrations [224–227] and reducing infrared emissivity [228–231].

Chen et al. [232] proposed an integrated sensing and communication (ISAC) system based on a spatiotemporal coding metasurface (STCM), enabling efficient coordination of communication and sensing functionalities through STCM technology, as shown in Fig. 13E. The research team introduced an encoding strategy where the metasurface can be dynamically configured using 2 different approaches. In one approach, the metasurface is divided into a spatiotemporal coding modulation region, which generates harmonics for sensing, and a spatial coding region, which adjusts the fundamental wave beam direction for communication. By dynamically allocating the area of these regions, resource utilization is optimized. Alternatively, a global spatiotemporal coding matrix (STC) is applied across the entire aperture, allowing simultaneous control of the fundamental wave propagation direction and the spatial distribution of harmonics. Specifically, by periodically modulating the metasurface elements using an STC, an incident fundamental wave (e.g., 10.3 GHz) is converted into multiple harmonic orders (e.g., ±1 to ±5 orders). The spatial distribution characteristics of these harmonics, such as amplitude and direction, are directly correlated with the DOA of the incident wave, providing a physical-layer information source for sensing. Experiments were conducted using a 16 × 16 unit, 2-bit reflective STCM prototype operating at 10.3 GHz, integrating PIN diodes to achieve 4-state phase modulation. Performance was validated using a software-defined radio platform (USRP). The results showed that the full-aperture scheme effectively suppressed the fundamental frequency component while achieving uniform harmonic distribution over −60° to +60°. In the adjustable partitioning scheme, the communication beam achieved a pointing accuracy of ±5°, and harmonic sensing remained unaffected by communication mode interference. DOA estimation based on an ANN exhibited an error of less than 3°. Even when handling QPSK modulated signals, the system maintained real-time beam adaptation, achieving reliable communication with a bit error rate (BER) of <10^−3^ and an EVM of <5% in both indoor and outdoor environments. This study offers new insights for the future development of dual-functional wireless networking technologies.

Conventional transmissive metasurfaces often rely on multilayer structures or external power sources, limiting optical transparency and flexibility in wave manipulation. To address these challenges, Sun et al. [233] proposed an intelligent microwave transmissive metasurface with optical sensing and transparency, enabling simultaneous microwave and optical signal transmission for cross-wavelength information transfer. As shown in Fig. 13F, the metasurface is designed on a PET substrate with a thickness of only 0.125 mm, offering excellent flexibility and optical transparency.

The structure consists of symmetrically concentric square-ring copper traces (with dimensions of 0.26λ @ 2.6 GHz) and integrates 4 photosensitive resistors (SG3624). By leveraging variations in light intensity, the resistors dynamically adjust their resistance (ranging from 200 kΩ to 50 Ω), altering the impedance characteristics of the unit cells to achieve dynamic control of microwave transmission amplitude without the need for additional optical components or power supplies. Experimental results demonstrated that the metasurface supports full-polarization (TE/TM) incidence with a maximum incident angle of 60° and can conform to complex surfaces such as cylindrical and wavy structures. When the optical path is blocked, the metasurface exhibits high microwave transmission efficiency at 2.78 GHz, with an S21 difference of up to 10 dB, whereas when the optical path is open, microwave transmission is suppressed, achieving an S21 difference of 15 dB, while allowing free passage of optical signals, validating the dual-channel adaptive switching capability. Additionally, in the absence of light, the metasurface facilitates efficient microwave transmission at 2.6 GHz, whereas under illumination, it effectively blocks microwave propagation. These characteristics indicate that the metasurface not only excels in both optical and microwave transmission but also offers rapid response to light intensity variations and flexible control over microwave transmission amplitude. This work provides a promising pathway for the development of hybrid optical–microwave communication systems and low-power sensing devices.

Li et al. [234] proposed an intelligent wireless power transfer framework based on a 2-bit compact reconfigurable transmissive metasurface router, addressing power supply challenges for IoT devices in complex environments, as shown in Fig. 13G. This framework consists of a planar wave feeder and a flat-panel 2-bit reconfigurable metasurface beam generator, forming a reconfigurable power router with a total thickness of only 0.8 wavelengths. By integrating deep learning-driven environmental sensors, the router enables target detection and localization, facilitating intelligent wireless power transfer in dynamic multi-target scenarios. Experimental results further demonstrated that the router is capable of simultaneously transmitting both wireless energy and information, highlighting its potential for next-generation smart wireless power transfer systems.

To further extend intelligent metasurfaces toward the deep integration of broadband communication and sensing, enabling the evolution from single-layer physical control to multifunctional system integration, Liu et al. [235] proposed a multifunctional RIS with significant application potential in both communication and sensing. As shown in Fig. 13H, the proposed RIS consists of a 20 × 20-unit array with a total thickness of only 0.8 wavelengths, substantially smaller than conventional designs. Each unit is equipped with 2 PIN diodes, enabling adjustable 2-bit local phase control with a reflection loss of less than 1.3 dB and a phase difference of 180°±30°, achieving a relative bandwidth of 41.3%. Test results demonstrated that the system can enhance signals by an average of 8 dB within the 15- to 21-GHz frequency range and successfully transmit 16-quadrature amplitude modulation signals at a symbol rate of 600 MHz.

In terms of sensing, the RIS is capable of detecting objects in the background and further enhances system capacity through a joint frequency and spatial diversity technique. Experimental results confirm that the RIS provides stable energy supply and information transmission for multiple dynamic targets in complex environments, highlighting its potential applications in IoT power delivery and 6G communication systems.

## Conclusion and Perspectives

### Trace biochemical sensing

With the widespread application of the THz wave in biosensing and medical diagnostics, THz metasurface-based biosensors have emerged as a prominent research focus. These sensors enable real-time monitoring of biomolecular interactions, structural changes, and concentration variations without direct contact with the sample, offering innovative solutions for early disease diagnosis and precision medicine. However, despite significant theoretical and experimental advances, THz metasurface biosensors still face a number of challenges that need to be addressed.

#### Improve the overall detection stability of the system

On the one hand, the resonance properties of the metasurface sensor can be significantly affected by fabrication errors and environmental disturbances, leading to resonance frequency drift and reduced sensitivity; on the other hand, the stability of the THz-TDS system itself also affects the overall sensitivity and accuracy of the detection system. It is equally important to achieve system optimization in aspects such as the selection of constituent materials for metasurface sensor, the design of microstructures, the fabrication process, and the stability of THz-TDS.

#### Develop THz metasurface sensors with dynamic tunability and specific recognition capabilities

Active regulation of resonance characteristics is achieved by introducing external stimulus response mechanisms such as electric fields, heat, light, or mechanical. THz metasurface sensors will be enabled to achieve multi-channel, multi-index, and even multi-dimensional spectral domain highly sensitive trace biochemical substance detection. In terms of specific recognition, by further optimizing and developing antibody-modified specific recognition interfaces, molecular feature absorption enhancement, and pixelated metasurface fingerprint spectrum reconstruction strategies, THz metasurface sensors will be promoted from label-free refractive index sensing to highly sensitive and repeatable specific recognition.

#### Development of intelligent THz metasurface sensing systems

The next-generation development of THz metasurface sensors needs to achieve the following three key goals: high integration, multifunctionality, and big data-drive. AI and deep-learning techniques should be further leveraged in THz metasurface research to automate microstructure design, signal denoising, feature extraction, material recognition, and concentration prediction. This will achieve an automatic closed loop from “structure design–signal acquisition–data analysis–analyte identification”, further promoting THz metasurface sensing technology to a new stage of intelligence and practicality.

### Microwave communication and sensing

Although theoretical research on metasurfaces has made notable progress, RIS industrialization still faces several challenges. The primary concern is hardware imperfections. While RIS-related algorithms have achieved major breakthroughs, non-ideal phase shifts, unit coupling, and manufacturing errors in physical implementations remain obstacles to efficient control strategies. As the RIS field matures, researchers should shift focus from theoretical system limits to practical deployability and stability. Developing low-cost, high-precision, and low-loss RIS units will enable cost-effective deployment solutions sooner.

Moreover, most theoretical frameworks remain confined to ideal scenarios, lacking extensive real-world validation. This highlights insufficient interdisciplinary collaboration and underscores the urgent need for standardized testing platforms. Empirical performance evaluations will be decisive for RIS’s real-world impact and commercial adoption. Based on the current research foundation, we believe that further innovations can be made in the following areas.

#### Efficient and highly integrated wireless communication system

Future research on RIS is expected to advance toward higher levels of integration, intelligence, and actuation. For example, based on the existing research and technology of reconfigurable smart surface (RIS), one or more components, such as power amplifier, adaptive control system, and ambient wave modulation module, can be added to make RIS highly integrated with multiple functions. Furthermore, RIS holds significant promise in the domain of secure communications. By incorporating techniques such as time-division and space-division multiplexing, beamforming-based encryption, and independent multi-channel control, RIS can offer flexible and robust physical-layer security solutions to mitigate information leakage and unauthorized interference. Within the 6G network framework, RIS is envisioned to serve as a novel, programmable, cooperative, and intelligently orchestrated infrastructure element, driving wireless communication systems toward greater efficiency, security, and intelligence.

#### Novel multi-functional intelligent sensor network

In the future, metasurface sensor networks can also integrate multimodal information such as vision, acoustics, and electromagnetics to achieve highly robust perception and intelligent collaborative decision-making in complex environments, significantly improving the system’s adaptability, intelligence level, and actual deployment value. For example, with the help of deep learning models or neural network control architectures, metasurfaces are expected to achieve autonomous learning and dynamic decision-making in the future, and thus have the ability to actively control mechanical and electronic systems. This ability to integrate perception and regulation gives it core application potential in future large-scale environmental perception and intelligent control systems.
